# Early Diverging and Core Bromelioideae (Bromeliaceae) Reveal Contrasting Patterns of Genome Size Evolution and Polyploidy 

**DOI:** 10.3389/fpls.2020.01295

**Published:** 2020-09-09

**Authors:** Juraj Paule, Sascha Heller, Jefferson Rodrigues Maciel, Raquel F. Monteiro, Elton M. C. Leme, Georg Zizka

**Affiliations:** ^1^ Department of Botany and Molecular Evolution, Senckenberg Research Institute and Natural History Museum, Frankfurt am Main, Germany; ^2^ Institute of Ecology, Evolution and Diversity, Goethe University, Frankfurt am Main, Germany; ^3^ Jardim Botânico do Recife, Prefeitura da Cidade do Recife, Recife, Brazil; ^4^ Department of Botany, Federal University of Rio de Janeiro, Rio de Janeiro, Brazil; ^5^ Marie Selby Botanical Gardens, Sarasota, FL, United States; ^6^ Rio de Janeiro Botanical Garden, Rio de Janeiro, Brazil

**Keywords:** bromeliads, chromosome number, climate, C-value, GC content, flow cytometry, phylogenetic signal, ploidy

## Abstract

The subfamily Bromelioideae is one of the most diverse groups among the neotropical Bromeliaceae. Previously, key innovations have been identified which account for the extraordinary radiation and species richness of this subfamily, especially in the so-called core Bromelioideae. However, in order to extend our understanding of the evolutionary mechanisms, the genomic mechanisms (*e.g.* polyploidy, dysploidy) that potentially underlie this accelerated speciation also need to be tested. Here, using PI and DAPI staining and flow cytometry we estimated genome size and GC content of 231 plants covering 30 genera and 165 species and combined it with published data. The evolutionary and ecological significance of all three genomic characters was tested within a previously generated dated phylogenetic framework using ancestral state reconstructions, comparative phylogenetic methods, and multiple regressions with climatic variables. The absolute genome size (2C) of Bromelioideae varied between 0.59 and 4.11 pg, and the GC content ranged between 36.73 and 41.43%. The monoploid genome sizes (Cx) differed significantly between core and early diverging lineages. The occurrence of dysploidy and polyploidy was, with few exceptions, limited to the phylogenetically isolated early diverging tank-less lineages. For Cx and GC content Ornstein–Uhlenbeck models outperformed the Brownian motion models suggesting adaptive potential linked to the temperature conditions. 2C-values revealed different rates of evolution in core and early diverging lineages also related to climatic conditions. Our results suggest that polyploidy is not associated with higher net diversification and fast radiation in core bromelioids. On the other hand, although coupled with higher extinction rates, dysploidy, polyploidy, and resulting genomic reorganizations might have played a role in the survival of the early diverging bromelioids in hot and arid environments.

## Introduction

Bromelioideae is the second most species-rich Bromeliaceae subfamily comprising 986 species as well as the most diverse subfamily concerning the number of genera (Gouda et al., 2020). The members of this subfamily occupy terrestrial, lithophytic, and epiphytic habitats in subtropical and tropical biomes in the Neotropics (Smith and Downs, 1979). The taxonomy of the subfamily is challenging as the present concepts still recognize polyphyletic groups as genera, especially in the case of the largest genus *Aechmea* (Schulte et al., 2009; Silvestro et al., 2014; Aguirre-Santoro et al., 2016). Moreover, the lack of resolution in phylogenetic studies and recent diversification (crown age of the extant lineages: 10.9 Ma; Silvestro et al., 2014) still limits our understanding of inter- and infrageneric evolutionary relationships (*e.g.* Schulte et al., 2005; Horres et al., 2007; Evans et al., 2015).

Nonetheless, the subfamily can be divided into two major groups: several informal early diverging tank-less lineages and mostly tank-forming core bromelioids (Schulte et al., 2009; Silvestro et al., 2014). The tank-less lineages are terrestrial or lithophytic usually characterized by succulent leaves with considerable water storage tissue and CAM photosynthesis, except the earliest diverging *Greigia*, *Ochagavia*, *Fascicularia*, as well as several other species from the Cryptanthoid complex and the genus *Fernseea*, which are C3 (Horres and Zizka, 1995; Silvestro et al., 2014; Crayn et al., 2015; Leme et al., 2017). In the tank-forming lineages of the core Bromelioideae, one large and/or several small tanks are formed by the tightly clasping leaf sheaths, which can thus impound up to several liters of water in larger plants (*e.g.* Cogliatti-Carvalho et al., 2010). The externally stored water is absorbed by leaf scales (tank-absorbing trichomes; Benzing, 2000; Males, 2016). The water bodies (phytotelmata) are also microhabitats and water source for a considerable range of animals and microorganisms (*e.g.* Wheeler, 1942; Frank and Lounibos, 2009). Interestingly, very few species among the core Bromelioideae lack the tank or have a rudimentary one (*e.g. Acanthostachys strobilacea, Araeococcus flagellifolius*), and only a few have been reported to have C3 instead of CAM photosynthesis (especially from genera *Nidularium* and *Ronnbergia*; Crayn et al., 2015).

Tank‐less Bromelioideae of the early diverging lineages includes over 200 species, whereas the core group comprises over 600 species (Gouda et al., 2020) and these two groups revealed contrasting evolutionary histories. It has been shown that the presence of a tank in core bromeliads is coupled with increased net diversification and five times lower extinction rates when compared to early diverging tank‐less clades (Silvestro et al., 2014). Hence, tank habit is considered an important key innovation and of great importance for the diversification of the species-rich epiphytic core group in the Atlantic and Amazonian rainforests. On the other hand, the majority of tank‐less species are found in dry habitats of Brazilian Cerrado and Caatinga or in the Andes as terrestrial or lithophytic xerophytes, and the higher extinction rate could be attributed to the absence of external water storage (tank) as well as to the periods of higher aridification during the Pleistocene climatic oscillations (Simon et al., 2009; Silvestro et al., 2014).

Genome size varies enormously among land plants, and the variation range is approximately 2,400-fold, which demonstrates the importance of ploidy level changes, the abundance of transposable elements, repetitive DNA, and chromosomal rearrangements in the plant evolution (Pellicer et al., 2018). This variation is considered to be the result of a combination of ecological, physiological, and morphological selection processes at the molecular level as genome size can impose phenotypic constraints on plant development, phenology, and ecological performance (Knight et al., 2005). In particular, monocot genomic diversity includes striking variation at many levels. Although the ancestral genome size of all monocots was small (1C = 1.9 pg), there have been notable increases in the rates of genome size-evolution particularly in Poales, to which the family Bromeliaceae belongs (Leitch et al., 2010). An increase in genome size by means of polyploidy has been repeatedly suggested for higher adaptive potential, which could be attributed to *e.g.* the increased genetic variability of polyploids, masking of mutations, gene redundancy, heterosis (*e.g.* Comai, 2005; Finigan et al., 2012). Indirect evidence for adaptive ecological significance of polyploidy was recently shown in *Fosterella* (Bromeliaceae: Pitcairnioideae) (Paule et al., 2017) and can be possibly assumed also for *Tillandsia* subg. *Diaphoranthema* (Till, 1992). Moreover, the GC content was considered to have an adaptive role due to differences in the physical properties of GC and AT base pairs (Šmarda and Bureš, 2012) and/or due to mode of endoreplication (Trávníček et al., 2019). In monocots, it has been demonstrated that higher GC contents are favored in cold and dry climates (Šmarda et al., 2014).

Within the subfamily Bromelioideae the dominant chromosome number is 2n = 2x = 50, similar to the whole family (Gitaí et al., 2014). Deviating chromosome numbers (2n = 32/34/36) were observed only in the genus *Cryptanthus* and *Hoplocryptanthus* and were attributed to dysploidy (Ramírez-Morillo and Brown, 2001; Gitaí et al., 2014; Cruz et al., 2020). Genome size (2C-values, Greilhuber et al., 2005) is small within the subfamily, ranging from 0.61 to 2.19 pg (Gitaí et al., 2014; Müller et al., 2019), and for GC content rather a broad interval ranging between 33.95 and 44.00% was reported (Favoreto et al., 2012; Šmarda et al., 2014). Polyploidy was so far documented only in two species of core Bromelioideae (*Aechmea eurycorymbus*, *Neoglaziovia variegata*; Cotias-de-Oliveira et al., 2000; Gitaí et al., 2014), but in several genera of early diverging Bromelioideae (*Ananas*: Collins and Kerns, 1936; Brown et al., 1997; *Bromelia*: Cotias-de-Oliveira et al., 2000; Gitaí et al., 2014; *Deinacanthon*: Gitaí et al., 2005). Nevertheless, the data on the genomic characters are scarce as chromosome numbers are known for *ca*. 16%, genome size for 13%, and GC content for 1% of the species only.

By adding substantial new data, we aim here 1) to analyze the distribution and potential evolutionary consequences of genome size and GC content variation as well as of the occurrence of polyploidy in the subfamily Bromelioideae. More specifically, using previously published phylogenetic framework combined with multiple regression analyses of genomic and climatic variables and model tests of genomic character evolution, we would like to examine whether 2a) previously confirmed fast radiation in the core tank-forming Bromelioideae (Silvestro et al., 2014) could be coupled with polyploidy and genomic rearrangements (assessed by divergent GC content and monoploid genome size (Cx); Trávníček et al., 2019) or, alternatively 2b) if the higher extinction rate in early diverging tank-less Bromelioideae is associated with these processes.

## Materials and Methods

### Plant Material

Altogether, 230 individual plants were sampled, covering 158 out of 986 (16.1%) currently recognized Bromelioideae species, 30 out of 37 Bromelioideae genera, and an additional 5 *Puya* outgroup species. We provide for the first time genome size for 127 and GC content estimates for 152 Bromelioideae species. The sampling covered 101 (including one published record) out of 114 species of the Bromelioideae phylogeny by Silvestro et al. (2014) and aimed representatively to cover the whole subfamily including so far underrepresented genera such as *Bromelia*. The studied material included 1 to 6 accessions per species. Taxonomic assignments and nomenclature are based on the Encyclopaedia of Bromeliads v4 (Gouda et al., 2020), except for the *Ananas* group, for which we applied the taxonomy as used by Matuszak-Renger et al. (2018). A list of all studied samples, their geographic origin, collection history, and herbarium voucher information is provided in **Supplementary Table 1**.

### Relative Genome Size, Absolute Genome Size, and GC Content Estimation

Relative genome sizes (RGS) were estimated by flow cytometric analyses of fresh leaves using a CyFlow space (Partec, Münster, Germany) fitted with a high power UV LED (365 nm). Leaf tissues of the analyzed sample and internal standard *Glycine max* cv. Polanka (2C = 2.50 pg) or *Pisum sativum* cv. Ctirad (2C = 9.09 pg) (Doležel et al., 1994; Doležel et al., 1998) were homogenized using a razor blade in a plastic Petri-dish containing 1 ml of ice-cold Otto I buffer (0.1 M citric acid, 0.5% Tween 20; Otto, 1990) to extract the nuclei. The suspension was filtered through Partec CellTrics^®^ 30 µm to remove tissue debris and incubated for at least 5 min at room temperature. Isolated nuclei in filtered suspension were stained with 1 ml of Otto II buffer (0.4 M Na_2_HPO_4_ × 12H_2_O) containing the AT-specific fluorochrome 4′,6-diamidino-2-phenylindole (DAPI, 4 µg·ml^−1^) and *β*-mercaptoethanol (2 µg·ml^−1^). The relative fluorescence intensity was recorded for 3,000 particles (nuclei) with one to three replicates per accession. RGSs expressed as sample/standard ratios were calculated from the means of fluorescence histograms visualized using the FloMax v2.4d software (Partec, Münster, Germany). Only histograms with coefficients of variation (CVs) < 5% for the G0/G1 peak of the sample were considered. In the case of CVs exceeding this threshold, the measurement was discarded, and the sample was reanalyzed.

Absolute genome sizes (2C-values) were estimated using the identical protocol as for RGS except for the staining solution, which consisted of 1 ml of Otto II buffer and intercalating propidium iodide (PI) and RNase IIA (both at final concentrations of 50 μg·ml^−1^). Fluorescence was induced by 30 mW green solid-state laser (532 nm) and fluorescence intensities of 5,000–10,000 nuclei per measurement were recorded. Two to eight replicate measurements of each sample were carried out on different days. The between-day variation caused by random instrument drift and/or non-identical sample preparation was low, and the difference between the maximum and minimum values of replicates did not exceed a 4% threshold.

The 2C-values were calculated by multiplying the sample/standard ratios with a known genome size of the standard *G. max*. *Pisum sativum* was used as a standard in five cases (**Supplementary Table 1**), in which the sample signal overlapped with the signal of *G. max*. The 2C-values estimated using the internal standard *P. sativum* were adjusted to those using *G. max* by multiplying the values by the coefficient of 3.772 which was based on 12 repeats of ratios among the two standards. Monoploid genome sizes (Cx-values; Greilhuber et al., 2005) were calculated for the species for which the chromosome number or ploidy level was known by dividing the 2C-values with respective ploidy. The estimation of GC content was based on a comparison of nuclei fluorescence stained with the DNA intercalating propidium iodide (PI ratio) and AT-specific DAPI (RGS) using the protocols and the GC content calculation tool by Šmarda et al. (2008). The base content of standard *G. max* (0.636) was extracted from Barow and Meister (2002).

### Literature Review

For the review of previously published chromosome numbers, genome size, and GC content estimates, taxa were critically assessed, and the nomenclature of Gouda et al. (2020) was followed. We retrieved 71 chromosome counts, which could be related to new or published genome size estimates, 54 chromosome counts for species present in the underlying phylogeny (**Supplementary Table 2**), and 43 genome size and 5 GC content estimates, which were included in further analyses (**Supplementary Table 3**).

### Data Analyses

Statistical analyses were performed in R v4.0.2 (R Core Team, 2020). The relationship between chromosome counts and 2C-values was assessed by Pearson’s correlation coefficient as well as linear regression. The differences between all 2C-values, monoploid genome sizes, and GC content estimates of early diverging and core bromeliads were tested using the non-parametric Wilcoxon rank-sum test (due to departure from normality assessed by Shapiro–Wilk normality test). For the comparison of the monoploid genome sizes, *Cryptanthus* and *Hoplocryptanthus* were excluded due to diverging presumably dysploid genomes. If several accessions per species were measured, those with higher 2C-value for each ploidy level were analyzed further using comparative methods. If a new and published 2C estimate was available, a newly generated data point was taken.

Comparative analyses were performed in R using phytools v0.6-60 (Revell, 2012) and OUwie v2.3 (Beaulieu et al., 2012) and maximum clade credibility chronogram of Silvestro et al. (2014) for all three genomic characters (2C, Cx, GC content). The phylogenetic signal was calculated as Pagel’s *λ* (Pagel, 1999) with a test of departure from *λ* = 0 by a likelihood-ratio test using function phylosig in phytools. To determine whether rates of genomic characters’ evolution differ among the early diverging and core clades, the fit of two Brownian motion (BM) and five Ornstein–Uhlenbeck (OU) models was compared using OUwie. Both BM and OU models estimate the rate of stochastic motion (*σ* ^2^). OU process allows the trait to fluctuate around an optimum value (*θ*) in parameter space with a strength of attraction (*α*) towards that optimum, while BM allows the trait to move equally to any parameter space. Models BM1 and BMS assign single and multiple rates (*σ* ^2^) of random drift. OU1 and OUM model single and multiple optima (*θ*) for different clades with a single *α* and *σ* ^2^. The remaining models assume either multiple *σ* ^2^ (OUMV), multiple *α* (OUMA), or both (OUMVA) among clades. When fitting models using OUwie, the starting value *θ*_0_ was dropped from the model and assumed to be distributed according to the stationary distribution of the OU process (default setting). The performance of each model was assessed by 1) confirming that the eigenvalues of the Hessian matrix were positive (Beaulieu et al., 2012) and 2) checking that the estimated optima (*θ*) of traits were not outside a plausible range. Only models passing these criteria were retained. The best-fitting model was selected using AIC weights based on the sample size‐corrected Akaike information criterion (AICc) using the function aic.w in phytools.

To assess the environmental correlates of the genomic characters, 19 climatic variables were extracted from the WorldClim v1.4 database (Hijmans et al., 2005) downloaded at the 30 seconds resolution for all Bromelioideae species based on the recently published species distribution ranges (Zizka et al., 2020). The shape of the refined distributional polygons was used as a ‘zonal feature’ to extract values of each Bioclim variable for all species using ArcGIS v10.0. Then, the mean of each climatic variable was calculated for every species. These mean values were used to explore climatic preferences of core and early diverging lineages by principal component analysis (PCA) implemented in function dudi.pca from the R package ade4 v1.4-14 (Chessel et al., 2004). For variable pairs with absolute correlation coefficients higher than 0.75 (**Supplementary Table 4**) only one, biologically more significant variable, was kept in order to not overemphasize the contribution of a particular climatic factor. Statistical differences between climatic niches of both groups were tested using the Wilcoxon rank-sum test of principal components. Relationships between genomic characters and climatic variables were further evaluated by multiple phylogenetic generalized least-squares (PGLS) (Grafen, 1989) based on a reduced genomic dataset (89 taxa) selected to match the phylogenetic tree and available distribution data. The PGLS analyses were carried out using the R package caper v1.0.1 (Orme et al., 2018) with the *λ* value estimated by maximum likelihood. Due to possible violation of PGLS assumptions, relationships between 2C and climatic variables were further evaluated by multiple linear regressions based on the whole genomic dataset matching the extracted distributions ranges (172 taxa).

Ancestral chromosome number reconstruction was carried out to assess the temporal dimension of polyploidization events. Estimates of ancestral chromosome numbers were inferred using maximum likelihood (ML) under the Markov k-state 1 (Mk1) parameter model, using the software Mesquite v2.74 (Maddison and Maddison, 2010). Mk1 implements a single parameter for the rate of change among any character state (Lewis, 2001). Different chromosome numbers were coded as categorical characters and proportional likelihood (PL) values were used to determine which ancestral state was the most likely. The ancestral states of monoploid genome size (Cx) and GC content were reconstructed using the maximum likelihood estimation (function fastAnc) and visualized on the phylogenetic tree using the function contMap in phytools.

## Results

### Genomic Characters

The genomes of the analyzed Bromelioideae species (**Supplementary Table 1**) were relatively small, ranging from 0.59 pg (*Orthophytum disjunctum* var. *viridiflorum*) to 4.11 pg (*Deinacanthon urbanianum*) for 2C and 0.27 pg (*Orthophytum compactum*) to 0.78 pg (*Greigia sphacelata*) for Cx-values. The genomes of five *Puya* species varied from 2C = 1 pg in *Puya densiflora* to 2C = 1.30 pg in *Puya ferruginea*. The differences between new and previously published 2C-values or among several accessions per species were low and did not exceed 5%, except cases in which the determination was provisional (“cf.”) or the differences could be attributed to polyploidy. The GC content ranged from 36.73 (*Deinacanthon urbanianum*) to 41.43% (*Aechmea filicaulis*), which represents a smaller range than previously reported by Favoreto et al. (2012). Hence, the published outlier GC content values were excluded from further analyses.

### Polyploidy and Dysploidy

**Figure 1:**
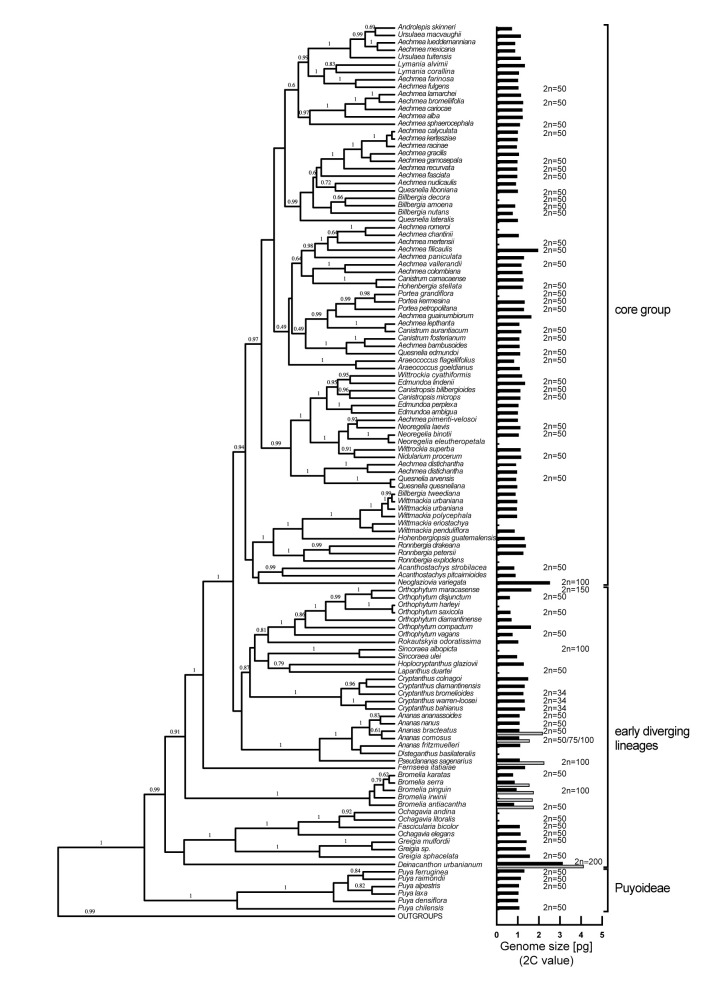
Phylogenetic tree of the subfamily Bromelioideae with the posterior probabilities estimated by BEAST above branches (adapted from Silvestro et al., 2014). Genome size (2C-value) is shown to the right of the tree as a black bar. The presence of a gray bar indicates intraspecific variation in genome size (*i.e.* ploidy variation).

To explore if genome size can be used as a proxy for ploidy level in Bromelioideae, published chromosome numbers were combined with available 2C-value data (**Figure 1**). After the exclusion of *Cryptanthus* and *Hoplocryptanthus* due to deviating chromosome numbers and presumed dysploid genomes, the correlation analysis showed a strong positive linear relationship and presented a robust linear model (Pearson’s r = 0.85, df = 61, CI_95%_ = 0.77, 0.91). Accordingly, when omitting the outliers for 2n = 50 (*Greigia sphacelata*, 2C = 1.56 pg; *Aechmea filicaulis*, 2C = 1.95 pg), we can assume diploidy for the majority of bromelioids with 2C < 1.37 pg (**Supplementary Figure 1**), although *Orthophytum* certainly represents a critical group with possible tetraploid 2Cs close to this threshold. In the case of polyploidy, a specific ploidy level was attributed based on the multiplication factor within a particular evolutionary lineage as shown *e.g.* for genus *Bromelia*, *Neoglaziovia variegata* or *Pseudananas sagenarius*, for which chromosome numbers are known (**Figure 1**, **Supplementary Table 1**). We identified several cases of infrageneric and intraspecific ploidy variation in the early diverging lineages such as *Ananas*, *Bromelia*, *Deinacanthon*, and *Pseudananas*, while diploids with only two exceptions (*Aechmea eurycorymbus*, *Neoglaziovia variegata*) were found in the core group (**Figure 1**, **Supplementary Table 1**). In three cases ploidy assignment was questionable (*Aechmea filicaulis*, *Aechmea guainumbiorum*, *Ronnbergia deleonii* (**Supplementary Table 1**) due to higher but not yet a multitude value of 2C within a presumably diploid lineage.

As mentioned above, dysploid chromosome numbers (2n = 32/34/36) were previously found in the genera *Cryptanthus* and *Hoplocryptanthus* of the early diverging group (Gitaí et al., 2014; Cruz et al., 2020). The genome size within the genus *Cryptanthus* is in our dataset relatively conserved (2C = 1.36 ± 0.09 pg) and we assume that also for the *Cryptanthus* and *Hoplocryptanthus* species without chromosome counts dysploid chromosome numbers can be expected based on previous chromosome count calibrations (Gitaí et al., 2014; Cruz et al., 2020).

### Ancestral Chromosome Number

**Figure 2:**
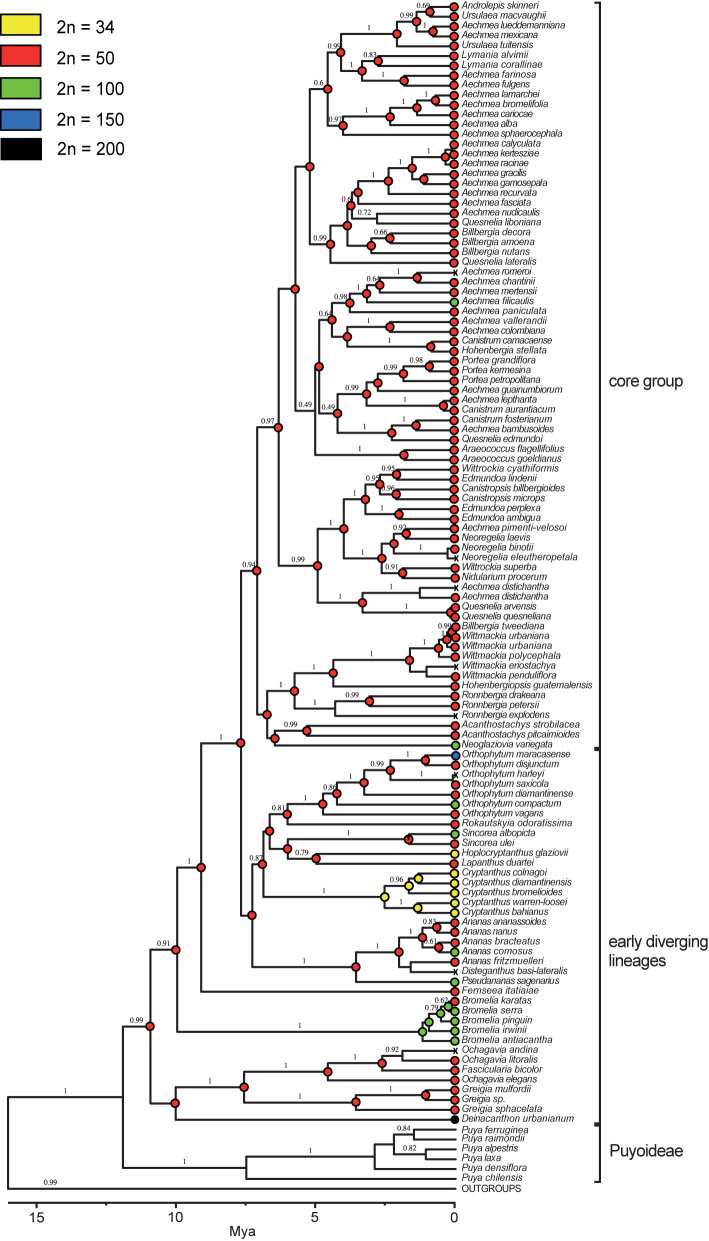
Ancestral chromosome number reconstructions of the subfamily Bromelioideae (adapted from Silvestro et al., 2014). Chromosome numbers of each accession (tips) and reconstructed proportional likelihood at each node are indicated by colored circles. “X”, missing data. The scale bar (Mya) below the tree represents time as reconstructed by Silvestro et al., 2014 and the values above branches are the posterior probabilities estimated by BEAST.

Using 2C-values as a proxy for ploidy level (see above) together with previously published chromosome numbers a reconstruction of ancestral chromosome numbers was carried for internal nodes of the Bromelioideae phylogenetic tree (**Figure 2**). The ancestral chromosome number inferred for the subfamily was 2n = 50 with a PL value of 0.99. For all but nine nodes a diploid ancestral chromosome number of 2n = 50 was inferred (PL = 0.99–1.00) as well. Nodes with a PL of a dysploid/polyploid ancestor were present in several lineages from the early diverging clades, namely *Bromelia*, *Cryptanthus*, *Sincoraea* (**Figure 2**).

### Genome Size Evolution

**Figure 3:**
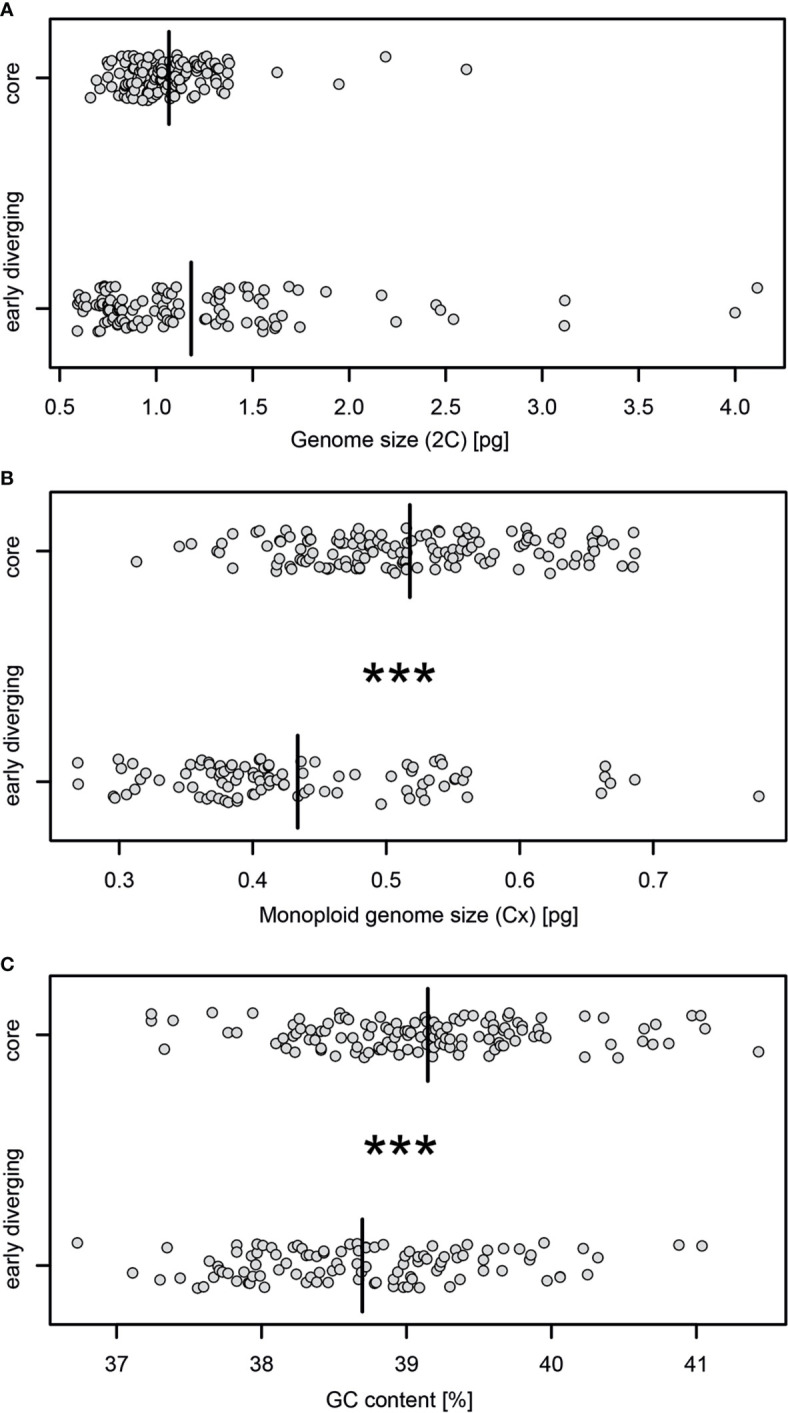
Stripchart comparisons of the **(A)** genome size estimates (2C), **(B)** monoploid genome size (Cx), **(C)** GC content between early diverging and core Bromelioideae. Asterisks indicate statistical significance based on the non-parametric Wilcoxon rank-sum test (***p ≤ 0.001), black bars indicate mean values of a particular variable and ploidy.

For the 2C-values of early diverging and core bromelioids insignificant differences were recovered (W = 7551.5, p = 0.4075), although the variance in the early diverging lineages was 6.5 times higher than in the core group (0.399 *vs* 0.061, **Figure 3A**). For the Cx-values (W = 3209, p < 0.001) (**Figure 3B**) as well as GC content (W = 4462, p < 0.001) (**Figure 3C**) significant differences were recovered between early diverging and core bromelioids. The phylogeny explains the distribution of 2C-values (*λ* = 0.842, p < 0.001), Cx-values (*λ* = 0.974, p < 0.001) as well as GC content (*λ* = 0.762, p < 0.01) (**Table 1**). When fitting multi-regime models of trait evolution, different models were suggested for studied three genomic characters. For 2C-values BMS model was best-fitting revealing 12 times higher stochastic motion around optimum in the early diverging lineages. For Cx-values OUMV model revealed diverging optima in both groups as well as three times higher stochastic motion in the early diverging lineages. For the GC content, the phylogenetic signal was confirmed by fitting the OU1 model (AICc weight = 0.61) suggesting adaptive potential towards a common optimum of both groups. However, AICc weights favored also OUM with a conditional probability of 0.29, but the difference in estimated optima was rather small (core 39.27 vs early diverging 39.11), which is in line with the OU1 as the best-fitting model (**Table 1**). The performance of fitted models that passed the criteria mentioned above is summarized in **Supplementary Table 5**. The Bromelioideae ancestral monoploid genome size was 0.56 pg, and the ancestral GC content was 39.48% (**Supplementary Figures 2** and **3**).

**Table 1:** Parameters estimated using best-fitted models of trait evolution for 2C, Cx, and GC content.

Genomic character/model	groups	*α*	*σ* ^2^	*θ* (S.E.)
2C/BMS	early diverging	NA	0.1188	1.6834 (0.4511)
	core	NA	0.0133	1.6834 (0.4511)
Cx/OUMV	early diverging	2.0411	0.1297	0.5067 (0.0374)
	core	2.0411	0.0397	0.5287 (0.0124)
GC/OU1	both	1.644	2.122	39.2234 (0.0838)

α, strength of attraction towards optimum; σ^2^, rate of stochastic motion; θ, optimum value; S.E., standard error.

### Climatic Niches and Regression Analyses

Climatic niches of early diverging and core lineages were compared by PCA (**Supplementary Figure 4**). Together, the first two axes (PC1, PC2) explained 61.90% of the total variance. PC1 (37.50% of the variance) corresponds to a gradient in temperature seasonality (bio4), the minimum temperature of the coldest month (bio6) and the precipitation of the driest month (bio14). Loadings of variables for PC2 (24.40% of the variance) correspond to variation in precipitation of the warmest quarter (bio18), the maximal temperature of the warmest month (bio5), and precipitation of the wettest month (bio13). A shift of 95% inertia ellipses of early diverging and core lineages recovered by the PCA suggested climatic differentiation (**Supplementary Figure 4A**), which was confirmed by significant differences for both PC1 (W = 81203, p < 0.001) and PC2 (W = 58594, p < 0.001). Hence, the species from early diverging lineages tend to occupy drier and colder habitats with higher temperature seasonality than species from core lineages.

Only temperature related variables were significantly associated with the genomic characters when considering multiple PGLS regressions. All temperature related variables (bio2, bio4, bio5, bio6) were significantly associated with Cx, and the model explained 20.26% of the variation (p < 0.05), similarly as for GC content (except bio4; multiple R^2^ = 0.2737, p < 0.001). No climatic variables were significantly associated with 2C-values using PGLS (**Supplementary Table 6**); however, non-phylogenetic multiple regression revealed a significant model (p = 0.004) explaining 11.65% of the variation.

## Discussion

Our study revealed strong contrasts between the genomes of core and early diverging lineages. This was supported by pairwise comparisons, by the strong phylogenetic signal as well as best-fitting models of evolution for all three studied genomic characters. A strong phylogenetic signal for 2C in Bromelioideae was also previously observed by Müller et al. (2019).

In the core group the vast majority of the studied species was considered diploid (2n = 2x = 50). The incidence of polyploidy was low, with only two polyploid species and three cases of ambiguous ploidy level among all studied species and published records. 2C-values showed smaller variance when compared to the early diverging lineages suggesting that the genomes of the core group are much more conserved. Thus, we conclude that polyploidy did not play a role in the fast radiation and increased diversification of the core group identified previously (Silvestro et al., 2014). Additionally, diploidy and genomic uniformity may play a role in gene flow among core lineages. It has been shown that interspecific, as well as intergeneric experimental hybridization within core Bromelioideae, is possible (*e.g.* Zhang et al., 2012; Souza et al., 2017), and rare natural hybridization events (Furtado et al., 2003; Wendt et al., 2008; Matallana et al., 2016) are important drivers of evolution in the group (Goetze et al., 2016). Hence, uniform genomes in terms of genome size and chromosome number could be considered favorable for gene flow as chromosomal and genomic uniformity usually facilitates chromosome pairing and recombination (*e.g.* Rieseberg, 2001).

On the contrary, in the early diverging group 12 dysploid and 21 polyploid individuals (out of 116 samples) and several cases of intraspecific ploidy variation (*e.g. Bromelia laciniosa*, *Deinacanthon urbanianum*, *Pseudananas sagenarius*) were observed. The genomes from the early diverging clades showed higher Cx as well as 2C variation when compared to the core group. Polyploidy evolved at least eight times independently, between the recent past and 1.67 Ma (95% HPD 0.44–3.52), whereas for dysploidy an origin at 2.53 Ma (95% HPD 1.27–4.22) was inferred (**Figure 2**; Silvestro et al., 2014). A second dysploidy event can be assumed for the genus *Hoplocryptanthus*. Interestingly, for some *Cryptanthus* and *Hoplocryptanthus* species (Ramírez-Morillo and Brown, 2001; Cruz et al., 2020) also “half-sized” genomes (*e.g.* 2C = 0.75-0.90 pg vs 2C = 1.28–1.66 pg) related to 2n = 34 were reported (**Supplementary Table 2**, **Supplementary Table 3**). This implies that within dysploid lineages further diverging chromosomal speciation processes and large genome rearrangements may be assumed.

It is striking that the taxa from the clade with more variable genomes are distributed mainly in hot and dry habitats of Brazilian Cerrado and Caatinga as terrestrial or lithophytic xerophytes (**Supplementary Figure 4**). Concerning the Cx-values, the OUMV model assuming different stochastic motion around different optima was suggested as the best-fitting (**Supplementary Table 5**), although both groups experienced a genomic contraction compared to the ancestrally reconstructed estimate (**Supplementary Figure 2**). The selection of OUMV supported significant differences in Cx between core and early diverging lineages, differential evolution of Cx in both groups as well as adaptive scenario driven by temperature as revealed by significant associations with temperature related climatic variables using PGLS (**Figure 3**, **Table 1**). Gain or loss of single chromosomes as well as deletion or proliferation of DNA are considered the main sources of Cx variation. So far there is no evidence for widespread aneuploidy in Bromelioideae, and we excluded dysploid lineages from the analysis. Hence, deletion or proliferation of DNA may be considered the sole source of recovered Cx variation most probably caused by an altered abundance of transposable elements and repetitive DNA. Higher stress-induced retrotransposon activity as a response to increased drought has been identified previously on a microscale in *Hordeum* (Kalendar et al., 2000) and retrotransposons in *Crucihimalaya himalaica*, which likely contributed to the adaptation to high altitude, proliferated shortly after the uplift and climatic change of the Himalayas from the Late Pliocene to Pleistocene (Zhang et al., 2019). In the early diverging lineages three times higher rate around the optimum Cx (*σ* ^2^) was revealed (**Table 1**), and we, therefore, assume differential and/or elevated activity of transposable elements either as a response to current or historical climate conditions.

Regarding GC content, our data revealed small but significant differences in the GC content of both groups, with slightly higher mean GC content in the core group. Interestingly, both groups reach similar maxima, and the phylogenetic signal was weaker when compared with 2C and Cx, indicating a weaker link with the separation by the group. The common evolution of GC in both groups was also confirmed by the best fit of the OU1 model with a single evolutionary optimum and adaptive consequences. Similarly, as for Cx, PGLS regression revealed significant associations of GC content with temperature related climatic variables, predicting higher GC for higher mean diurnal range (bio2) as well as temperature seasonality (bio4). Higher GC content was in monocots associated with increased tolerance and ability to grow in regions of extremely cold winters or experiencing at least some seasonal water deficiency (Šmarda et al., 2014). Although our dataset does not represent sharp seasonal climatic variation and the interval of the GC content estimates is relatively small, our results would be in line with the general pattern observed in monocots explained by the higher thermal stability of GC base pairs (Vinogradov, 2003; Šmarda and Bureš, 2012).

We could not relate the variability of Cx and GC content with any studied precipitation related variables and confirm previously suggested water deficiency as a potential evolutionary driver of genomic characters. In terrestrial tank bromelioids such as *Aechmea bromeliifolia*, *A. nudicaulis*, or *Neoregelia cruenta* the water could be stored even over longer dry periods (Lopez and Rios, 2001). Accordingly, relationships with precipitation variables could suffer from a certain bias as low precipitation does not necessarily point towards drought stress due to the water storage capacity in the tank, which buffers against temperature-limited soil water availability (Benzing, 2000).

As revealed by the best-fitting BMS model, early diverging lineages revealed 12 times higher rate of stochastic motion around optimum 2C than the core lineages, which supports differential 2C evolution of both groups. Previously, the best fit to a single-optimum OU model was suggested for Bromelioideae (Müller et al., 2019). However, only single regime models have been considered in that study, and it has been shown that the OU model can be incorrectly favored over simpler models (Cooper et al., 2015). Interestingly, no significant associations of 2C with the climatic data were recovered, which suggests that the current climate is not a good predictor for 2C. Due to polyploidy, 2Cs of closely related species are not necessarily more similar, which might disturb some of the PGLS assumptions. Using a non-phylogenetic multiple regression (**Supplementary Table 6**), we recovered a significant multiple regression model (p < 0.01) with 11.65% explained variation. Accordingly, together with the prevalence of polyploids in early diverging group and 12 times higher rate of evolution, we assume that polyploidy in early diverging Bromelioideae plays a role in surviving in hot and dry climatic conditions as often assumed also in other plant groups (*e.g.* Manzaneda et al., 2012; Pyšek et al., 2018). However, to further test the associations with current climate a more comprehensive phylogenetic framework and/or additional distribution data would be advantageous as several polyploid 2C-values were missing in the analyses.

The relationship between polyploidy and harsher habitats has been hypothesized in many plant groups (Finigan et al., 2012; Manzaneda et al., 2012; Pyšek et al., 2018) and can be explained by increased heterozygosity, higher levels of diversity (*i.e.* higher number of alleles), and tolerance against increased levels of selfing or robustness of genomes against mutations (Comai, 2005). Here we additionally assessed Cx and GC content, which revealed patterns compatible with those hypothesized for polyploids. Interestingly, an analogical pattern as revealed for the whole subfamily was already observed within the early diverging genus *Orthophytum*. The polyploid *Orthophytum* species are closely associated with xeric microhabitats and possess xeromorphic traits (*e.g.* coriaceous, densely lepidote leaves) while the majority of diploid taxa were found in more mesic to wet microhabitats (Louzada et al., 2010). *Orthophytum* seems particularly interesting as it represents the strongest genomic contraction from the ancestral monoploid genome size 0.56 pg (**Supplementary Figure 1**), showing the smallest monoploid genomes in the subfamily (**Supplementary Table 1**) as well as polyploidy. Associations between genome size and different habitat preferences were also hypothesized for dysploid Cryptanthoid complex, relating bigger genomes of the genus *Cryptanthus* to moist Atlantic rainforest and smaller genomes of *Hoplocryptanthus* to dry campos rupestres (Cruz et al., 2020). Moreover, cytotypes in the genus *Fosterella* (Bromeliaceae: Pitcairnioideae) were found to be ecologically differentiated, showing that polyploids preferentially occupy colder habitats with high temperature seasonality (Paule et al., 2017) and members of polyploid *Tillandsia* subgen. *Diaphoranthema* (Tillandsioideae) were considered for high stress tolerance as they are found in more isolated and climatically extreme areas than any other *Tillandsia* species (Till, 1992).

The Quaternary climatic oscillations are considered key events in biodiversity diversification. Particularly in South America, the climatic oscillations led to a series of contractions and expansions of forest and non-forest vegetation as well as periods of aridification resulting in nowadays Cerrado and Caatinga (*e.g.* Simon et al., 2009; Werneck et al., 2012). Due to the estimated origin of the dysploidy in *Cryptanthus* at app. 2.53 Ma as well as of the earliest polyploidization events in *Bromelia* at app. 1.18 Ma most of the identified genomic alterations in the early diverging lineages could be associated with the Pleistocene climatic changes. Accordingly, the following processes or their combination can be assumed in the Quaternary history of Bromelioideae: 1) Climatic changes may have promoted repeated secondary contacts among otherwise geographically isolated Bromelioideae lineages resulting in hybridization and allopolyploidy. The combination of different genomes in these lineages might have further added towards an adaptive potential (Barker et al., 2016) and supported the survival of the early diverging bromelioids in the arid environments. A similar scenario can be assumed for other polyploid taxa from Cerrado such as *Mimosa* (Fabaceae; Morales et al., 2018) and *Eriotheca* (Malvaceae; Mendes-Rodrigues et al., 2019). However, the abundance of polyploidy and climatic preferences of polyploids in Cerrado and Caatinga remain to be tested across several taxa. 2) Climatic oscillations and/or other environmental factors could have triggered the formation of unreduced gametes as previously shown in *e.g. Solanum* (McHale, 1983). 3) Allopatric and parapatric speciation was triggered by dysploidy and polyploidy leading to reproductive isolation and the origin of polyploid and dysploid taxa such as *e.g. Deinacanthon urbanianum* or genus *Cryptanthus*, respectively. 4) Finally, it has been demonstrated that polyploids and plants with larger genomes are selectively disadvantaged under limited availability of environmental nitrogen and phosphorus (Šmarda et al., 2013; Guignard et al., 2016). Limited availability of both nutrients was exemplified in tank-forming epiphyte *Werauhia sanguinolenta* (Tillandsioideae) (Wanek and Zotz, 2011). Hence, nutrient shortage in almost exclusively tank-forming core Bromelioideae could be considered another factor contributing towards dominant diploidy in this group.

## Conclusion

The genomes in the core Bromelioideae were revealed to be strikingly uniform concerning both ploidy as well as monoploid genome size. Hence, polyploidy and genomic reorganizations are not associated with higher net diversification and speciation in core bromelioids. On the contrary, the early diverging lineages revealed a higher incidence of polyploidy, presumed dysploidy as well as higher variation in the monoploid genome size. For Cx and GC content Ornstein–Uhlenbeck models outperformed the Brownian motion models suggesting adaptive potential linked to the temperature conditions. 2C-values revealed different rates of evolution in core and early diverging lineages also related to climatic conditions. The origins of polyploidy in the subfamily could be followed back to the Pleistocene and could be most probably attributed to the dynamic climatic conditions in the areas of today’s Cerrado and Caatinga. Accordingly, although coupled with higher extinction rates, polyploidy and genomic reorganizations might have played a role in the survival of the early diverging bromelioids in the arid environments.

## Data Availability Statement

All datasets presented in this study are included in the article/**Supplementary Material**.

## Author Contributions

JP and GZ conceived the study. EL, RM, and JM provided plant material and taxonomic determinations. JP, SH, RM, and JM carried out the experiments. JP, SH, and JM analyzed the data and produced the figures. JP drafted the manuscript with contributions from all authors. All authors approved the final version of the manuscript.

## Funding

This work was supported by the research funding program “LOEWE—Landesoffensive zur Entwicklung wissenschaftlich-ökologischer Exzellenz” of Hesse’s Ministry of higher education; the “Freunde und Förderer” of Goethe-University Frankfurt; Paul Ungerer-Stiftung; German Research Foundation [DFG Zi 557/7-1, Schu2426/1-1]; DAAD (Deutscher Akademischer Austauschdienst), CNPq (Conselho Nacional de Desenvolvimento Científico e Tecnológico); CAPES (Coordenação de Aperfeiçoamento de Pessoal de Nível Superior) in scope of the programs PROTAX and PROBRAL, respectively; and FAPERJ (Fundação de Amparo à Pesquisa do Estado do Rio de Janeiro).

## Conflict of Interest

The authors declare that the research was conducted in the absence of any commercial or financial relationships that could be construed as a potential conflict of interest.
