# Supplementary material for: Early Diverging and Core Bromelioideae (Bromeliaceae) Reveal Contrasting Patterns of Genome Size Evolution and Polyploidy
Source: Front Plant Sci. 2020 Sep 9;11:1295. doi: 10.3389/fpls.2020.01295 (PMC7509451; doi:10.3389/fpls.2020.01295)

Supplementary Figure 1. Fitted line plot of chromosome numbers (2n) and genome size (2C) from the subfamily Bromelioideae excluding genera *Cryptanthus* and *Hoplocryptanthus*.

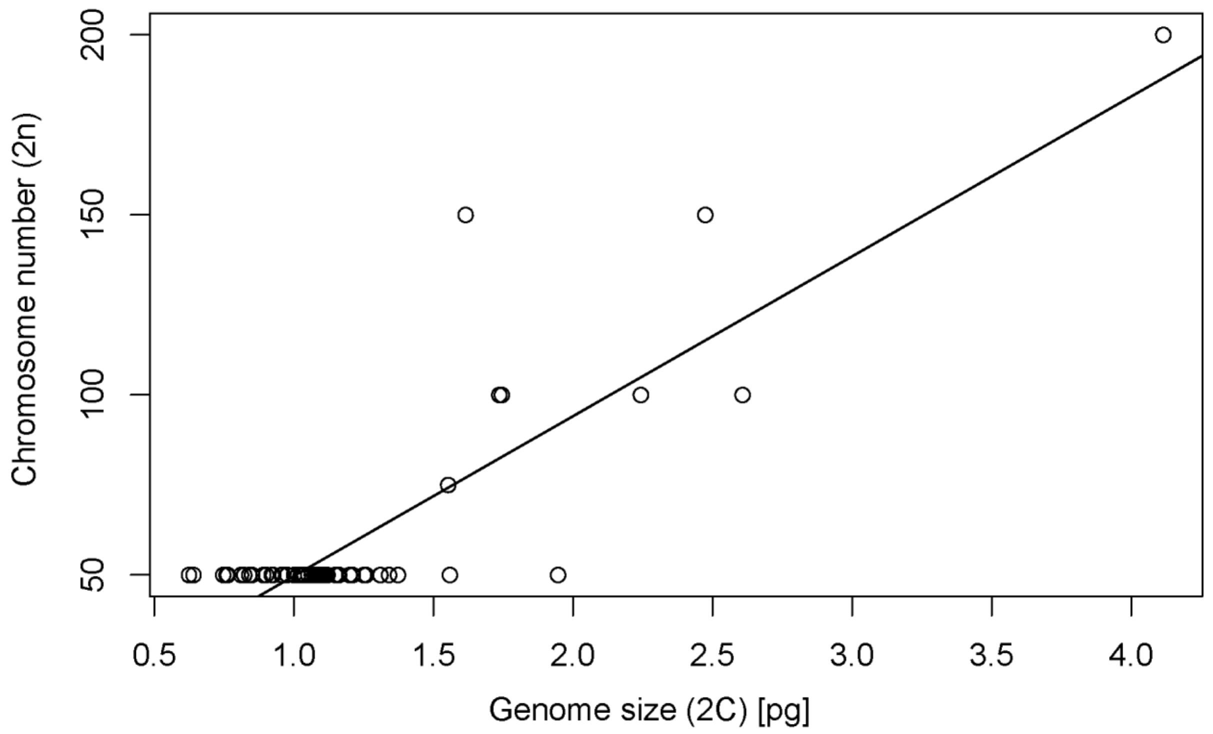

Supplement: Supplementary file 1 [file DataSheet_1.pdf]
