# Supplementary material for: Early Diverging and Core Bromelioideae (Bromeliaceae) Reveal Contrasting Patterns of Genome Size Evolution and Polyploidy
Source: Front Plant Sci. 2020 Sep 9;11:1295. doi: 10.3389/fpls.2020.01295 (PMC7509451; doi:10.3389/fpls.2020.01295)
Supplement: Supplementary file 3 [file DataSheet_3.pdf]

Supplementary Figure 3. Ancestral state reconstruction of GC content. A – colored bar left down indicates the interval of the GC content values, branches of the tree are colored accordingly, B – GC values reconstructed for nodes are shown in light blue rectangles.

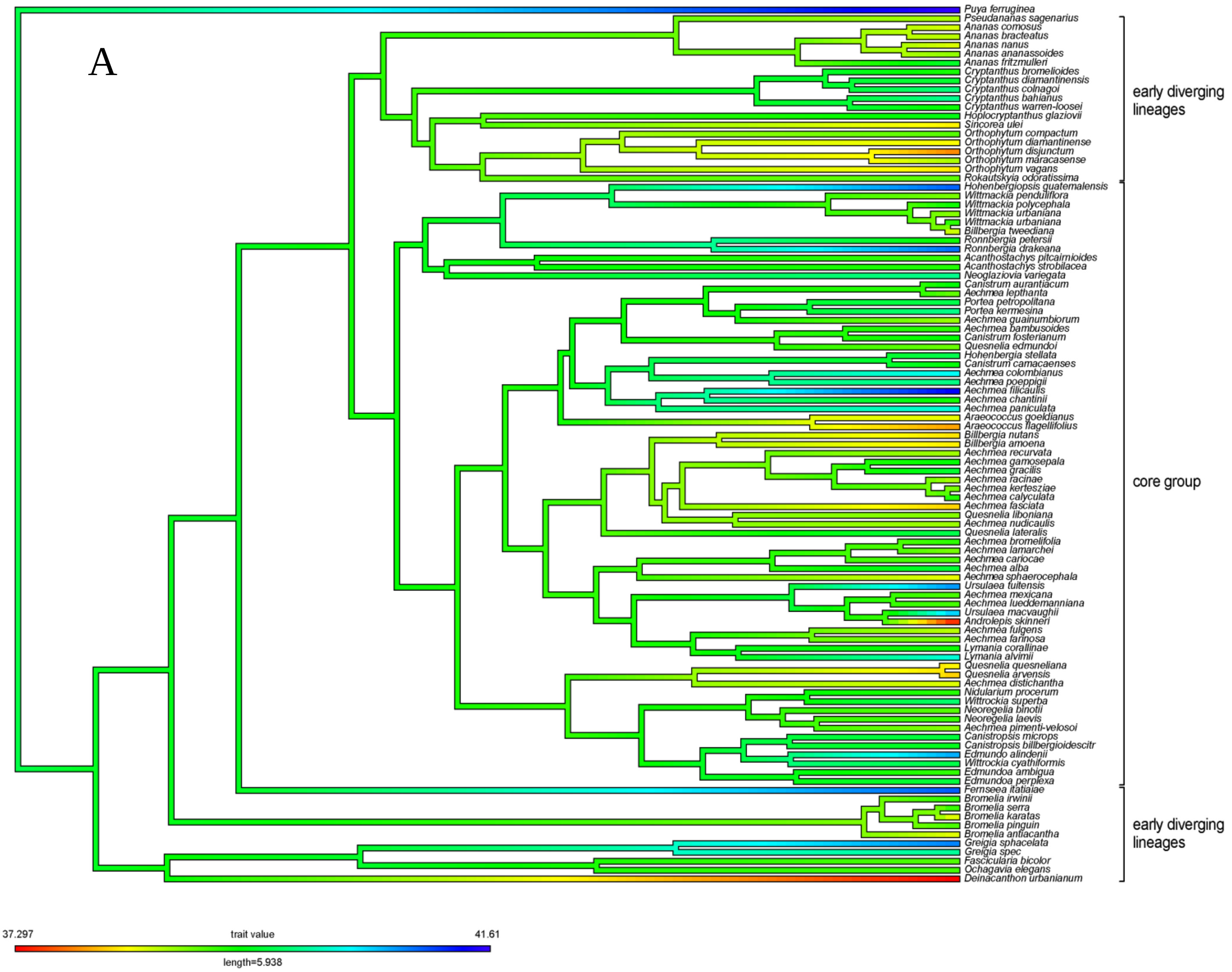

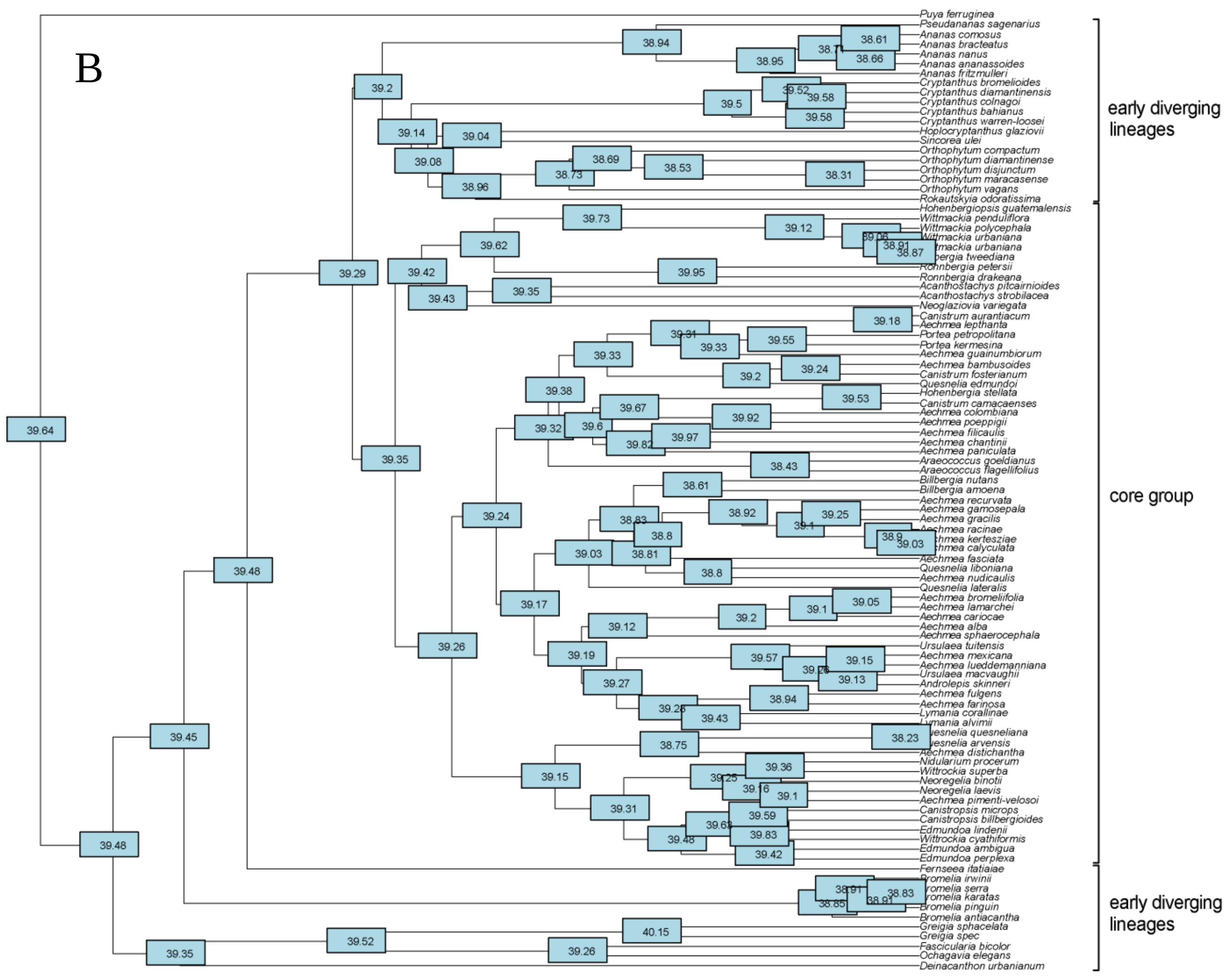
B
Puya ferruginea
Pseudananas sagenarius
Ananas comosus
Ananas bracteatus
Ananas nanus
Ananas ananassoides
Ananas fritzmulleri
Cryptanthus bromelioides
Cryptanthus diamantinensis
Cryptanthus colnagoi
Cryptanthus bahianus
Cryptanthus warren-loosei
Hoplocryptanthus glaziovii
Sincorea ulei
Orthophytum compactum
Orthophytum diamantinense
Orthophytum disjunctum
Orthophytum maracasense
Orthophytum vagans
Rokautskyia odoratissima
Hohenbergiopsis guatemalensis
Wittmackia penduliflora
Wittmackia polycephala
Wittmackia urbaniana
Ronnbergia petersii
Ronnbergia drakeana
Acanthostachys pitcairnioides
Acanthostachys strobilacea
Neoglaziovia variegata
Canistrum aurantiacum
Aechmea lepthanta
Portea petropolitana
Portea kermesina
Aechmea guainumbiorum
Aechmea bambusoides
Canistrum fosterianum
Quesnelia edmundoi
Hohenbergia stellata
Canistrum camacaenses
Aechmea colombiana
Aechmea poeppigii
Aechmea filicaulis
Aechmea chantinii
Aechmea paniculata
Araeococcus goeldianus
Araeococcus flagellifolius
Billbergia nutans
Billbergia amoena
Aechmea recurvata
Aechmea gamosepala
Aechmea gracilis
Aechmea racinae
Aechmea fasciata
Quesnelia liboniana
Aechmea nudicaulis
Quesnelia lateralis
Aechmea bromeliifolia
Aechmea lamarchei
Aechmea cariocae
Aechmea alba
Aechmea sphaerocephala
Ursulaea tuitensis
Aechmea mexicana
Aechmea lueddemanniana
Ursulaea macvaughii
Androlepis skinneri
Aechmea fulgens
Aechmea farinosa
Lymania corallinae
Lymania alvimii
Aechmea distichantha
Nidularium procerum
Wittrockia superba
Neoregelia binotii
Neoregelia laevis
Aechmea pimenti-velosoi
Canistropsis microps
Canistropsis billbergioides
Edmundoa lindenii
Wittrockia cyathiformis
Edmundoa ambigua
Edmundoa perplexa
Fernseea itatiaiae
Bromelia pinguin
Bromelia antiacantha
Greigia sphacelata
Greigia spec
Fascicularia bicolor
Ochagavia elegans
Deinacanthon urbanianum
early diverging lineages
core group
early diverging lineages
