# Supplementary material for: Early Diverging and Core Bromelioideae (Bromeliaceae) Reveal Contrasting Patterns of Genome Size Evolution and Polyploidy
Source: Front Plant Sci. 2020 Sep 9;11:1295. doi: 10.3389/fpls.2020.01295 (PMC7509451; doi:10.3389/fpls.2020.01295)

Supplementary Figure 4. Scatterplot showing results from principal component analysis (PCA) of the climatic variables extracted for distribution ranges of Bromelioideae species extracted from Zizka et al. (2020). The first two axes explain 37.50 and 24.40% of the variance among the 7 climatic variables. (A) Early diverging (black) and core (green) lineages labelled inside the 95% inertia ellipses. (B) Biplot of objects and variables, in which arrows represent direction and magnitude of effects of the environmental variables.

A

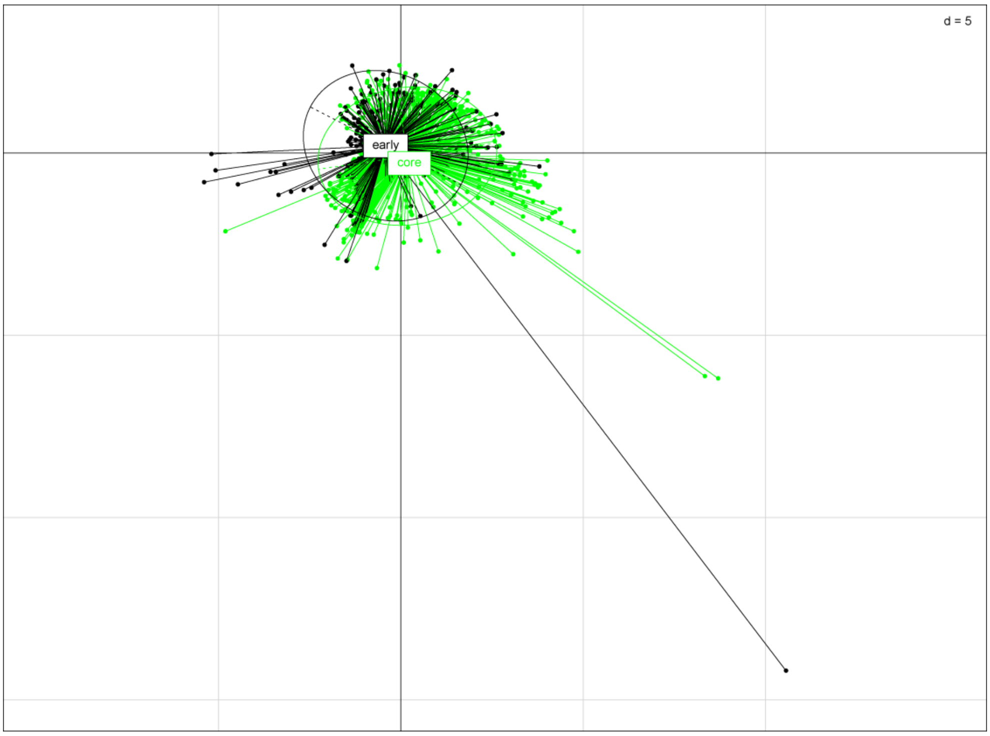

B

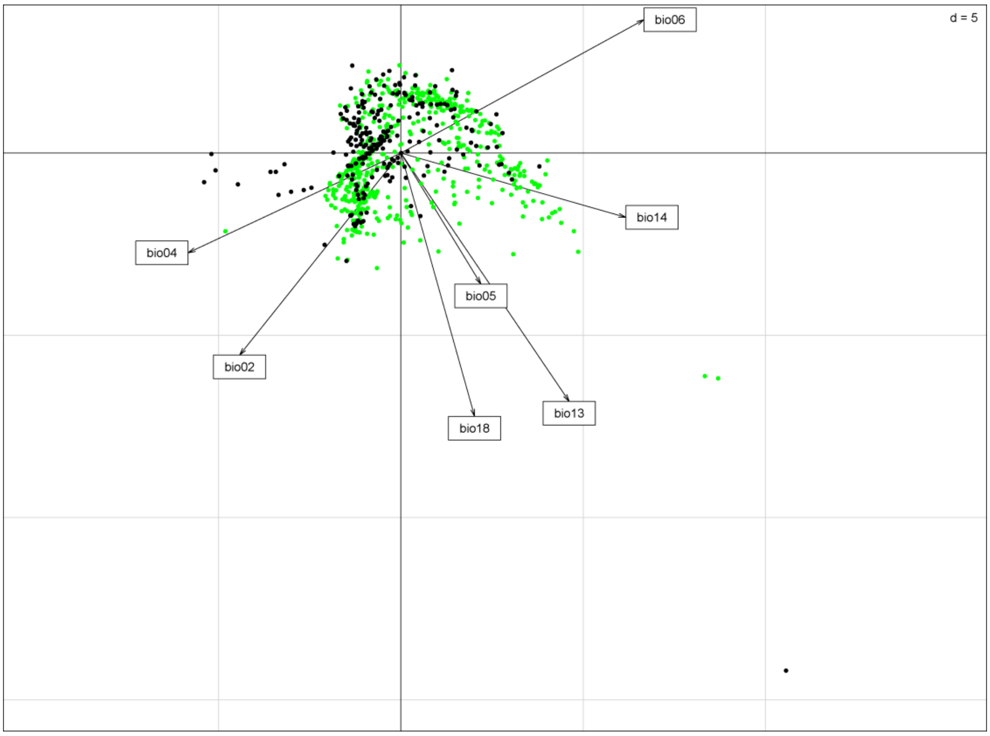

Supplement: Supplementary file 4 [file DataSheet_4.pdf]
