# Supplementary material for: Early Diverging and Core Bromelioideae (Bromeliaceae) Reveal Contrasting Patterns of Genome Size Evolution and Polyploidy
Source: Front Plant Sci. 2020 Sep 9;11:1295. doi: 10.3389/fpls.2020.01295 (PMC7509451; doi:10.3389/fpls.2020.01295)
Supplement: Supplementary file 6 [file Table_2.pdf]

Supplementary Table 2. Previously published chromosome numbers in the subfamily Bromelioideae and Puyoideae used in this study. Numbers in brackets do not follow the genus pattern and/or are regarded as approximate by the author (indicated by "≈") and were not considered in the statistical analyses. Nomenclature follows Gouda et al. (cont. updated). Names used in original publications are given in square brackets.

**BROMELIOIDEAE**			
**Taxon**	**n**	**2n**	**References**
Acanthostachys Link			
A. strobilacea (Schult. & Schult.f.) Klotzsch		50	Lindschau, 1933
Aechmea Ruiz & Pav.			
A. aquilega (Salisb.) Griseb.		50	Cotias-de-Oliveira et al. 2000; Gitaí et al., 2014
A. bracteata (Sw.) Griseb.		50	Lindschau, 1933; Gitaí et al., 2014
A. bromeliifolia (Rudge) Baker [also published as *Macrochordion tinctorium* De Vriese and *Aechmea conspicuiarmata* Baker]		≈50<br>50	Taylor, 1925<br>Lindschau, 1933; Gitaí et al., 2005; Brown et al., 1997
A. calyculata (E.Morren) Baker	25	50	Palma-Silva, 2003; Palma-Silva et al., 2004
A. coelestis (K.Koch) E.Morren	 25	50 	Lindschau, 1933<br>Marchant, 1967
A. eurycorymbus Harms		(≈88)	Gitaí et al., 2014
A. fasciata (Lindl.) Baker<br>*A. fasciata* var. *fasciata*	25<br>25		Marchant, 1967<br>Marchant, 1967
A. fendleri André ex Mez		50	Gitaí et al., 2014
A. filicaulis (Griseb.) Mez	25		Marchant, 1967; Brown et al., 1984
A. fulgens Brongn.<br>*A. fulgens* var. *fulgens*<br>*A. fulgens* var. *discolor* (C.Morren) Brongn. ex Baker	 <br>25 	50<br>50<br><br>50	Marchant, 1967<br>Cotias-de-Oliveira et al., 2004<br>Lindschau, 1933<br>Brown et al., 1997
A. gamosepala Wittm.		50	Palma-Silva 2003; Palma-Silva et al., 2004
A. mertensii (G.Mey.) Schult. & Schult.f.		≈50	Marchant, 1967
A. ornata (Gaudich.) Baker [also published as *Aechmea hystrix* E.Morren]		(54)<br>50	Lindschau, 1933<br>Gitaí et al., 2014
A. recurvata (Klotzsch) L.B.Sm.		50	Ceita et al., 2008
A. sphaerocephala (Gaudich.) Baker		50	Gitaí et al., 2014
A. vallerandii (Carrière) Erhardt, Götz & Seybold [published as *Streptocalyx poeppigii* Beer]	25		Marchant, 1967
A. victoriana L.B.Sm.	25		Marchant, 1967
A. weilbachii Didr.	25		Marchant, 1967
Ananas Mill.			For a review of A*nanas* chromosome numbers please see Matuszak-Renger et al., 2018; Online Resource 7
Araeococcus Brongn.			
A. flagellifolius Harms		50	Brown et al., 1997
Billbergia Thunb**.**			
B. amoena (Lodd.) Lindl. [published as *Billbergia speciosa* Thunb.]		(54)	Lindschau, 1933
B. decora Poepp. & Endl.		50	Marchant, 1967

B. euphemiae E.Morren<br>*B. euphemiae* var. *euphemiae*		50<br>50	Sharma and Gosh, 1971<br>Cotias-de-Oliveira et al., 2004
B. horrida Regel var. *tigrina* hort. ex Baker		50	Gitaí et al., 2014
B. nutans H.Wendl. ex Regel var. *nutans* [published as *Billbergia bonplandiana* Gaudich. ex Mez, *Billbergia minuta* Mez]	 25	(54)	Lindschau, 1933<br>Marchant, 1967
B. pallidiflora Liebm.		50	Gitaí et al., 2014
B. pyramidalis (Sims) Lindl.<br><br>*B. pyramidalis* var. *pyramidalis*<br>*B. pyramidalis* var. *striata* M.B.Foster	 25	(54)<br><br>50<br>50	Lindschau, 1933<br>Marchant, 1967<br>McWilliams, 1974<br>McWilliams, 1974
Bromelia L.			
B. antiacantha Bertol.		100	Gitaí et al., 2014
B. goeldiana L.B.Sm.		94	Lin et al., 1987
B. karatas L. [also published as *Bromelia plumieri* (E. Morren) L.B.Sm.]		50<br>50+1–2B	Éder-Silva et al., 2007<br>Cotias-de-Oliveira et al., 2000
B. laciniosa Mart. ex Schult. & Schult.f.		≈150	Cotias-de-Oliveira et al., 2000; Gitaí et al., 2005
B. pinguin L. [also published as *Bromelia fastuosa* Lindl.]	48	 ≈96–100	Collins and Kerns, 1931<br>Lindschau, 1933
Canistropsis Mez			
C. billbergioides (Schult. & Schult.f.) Leme		50	Bellintani et al., 2005
C. microps (E.Morren ex Mez) Leme [also published as *Aregelia microps* (E.Morren ex Mez) Mez]		(≈124–126)<br>50	Lindschau, 1933<br>Bellintani et al., 2005
Canistrum E. Morren			
C. aurantiacum E.Morren		50	Weiss, 1965
C. fosterianum L.B.Sm.		50	Bellintani et al., 2005
Cryptanthus Otto & A.Dietr.			
C. acaulis (Lindl.) Beer.	 17<br>(≈17)	36<br><br><br>34	Lindschau, 1933<br>Matsuura and Sutô, 1935<br>Marchant, 1967<br>Ramiréz-Morillo and Brown, 2001
C. bahianus L.B.Sm.	 <br>17	34+1–4B<br>34+1–3B	Cotias-de-Oliveira et al., 2000<br>Gitaí et al., 2014<br>Marchant, 1967
C. beuckeri E.Morren		(54)<br>≈34<br>34	Lindschau, 1933<br>Marchant, 1967<br>Bellintani et al., 2005
C. bromelioides Otto & A.Dietr.		34	Sharma and Gosh, 1971
C. marginatus L.B.Sm.		32	Gitaí et al., 2014
C. praetextus E. Morren ex Baker		34<br>32+1–2B	Sharma and Gosh, 1971<br>Gitaí et al., 2014
C. warren-loosei Leme		34	Ceita et al., 2008
Deinacanthon Mez			
D. urbanianum (Mez) Mez		(≈160)	Gitaí et al., 2005
Edmundoa Leme			
E. lindenii (Regel) Leme<br>*E. lindenii* var. *rosea* (E.Morren) Leme [published as *Canistrum roseum* E.Morren]		50<br>50	Gitaí et al., 2014<br>Lindschau, 1933
Fascicularia Mez			

F. bicolor (Ruiz & Pav.) Mez subsp. *bicolor*<br>*F. bicolor* subsp. *canaliculata* E.C.Nelson & Zizka		50<br>50	Gitaí et al., 2005<br>Gitaí et al., 2005
Greigia Regel			
G. spec. nov. (aff. *G. mulfordii* L.B.Sm. var. *macrantha* L.B.Sm.)		50	Gitaí et al., 2005
G. sphacelata (Ruiz & Pav.) Regel		50	Gitaí et al., 2005
Hohenbergia Schult.f.			
H. stellata Schult. & Schult.f.		50	Cotias-de-Oliveira et al., 2000
Hoplocryptanthus (Mez) Leme, S. Heller & Zizka			
H. schwackeanus (Mez) Leme, S. Heller & Zizka [published as *Cryptanthus schwackeanus* Mez]		34	Ramiréz-Morillo and Brown, 2001
Lapanthus Louzada & Versieux			
L. duartei (L.B. Smith) Louzada & Versieux [published as *Orthophytum supthutii* E.Gross & Barthlott]		50	Louzada et al., 2010
Neoglaziovia Mez			
N. variegata (Arruda) Mez		100	Cotias-de-Oliveira et al., 2000; 2004; Gitaí et al., 2014
Neoregelia L.B.Sm.			
N. binotii (E.Morren) L.B.Sm. [published as *Aregelia binotii* (E.Morren) Mez]	25, (27)	(54)	Lindschau, 1933
N. carolinae (Beer) L.B.Sm. [also published as *Aregelia carolinae* (Beer) Mez]		(54)<br>50	Lindschau, 1933<br>Bellintani et al., 2005
N. laevis (Mez) L.B.Sm. [published as *Neoregelia laevis* (Mez) L.B.Sm. var. *albomarginata*]		50	Cotias-de-Oliveira et al., 2004
Nidularium Lem.			
N. amazonicum (Baker) Linden & E.Morren ex Lindm. [published as *Canistrum amazonicum* (Baker) Mez]		50	Weiss, 1965
N. innocentii Lem. [published as *Nidularium lineatum* Mez]<br>*N. innocentii* var. *innocentii* Lem.	25	(54)<br><br>50	Lindschau, 1933<br><br>Ceita et al., 2008
N. procerum Lindm.		50	Cotias-de-Oliveira et al., 2004
Ochagavia Phil.			
O. elegans Phil.		50	Gitaí et al., 2005
O. litoralis (Phil.) Zizka, Trumpler & Zöllner		50	Gitaí et al., 2005
Orthophytum Beer			
O. disjunctum L.B.Sm.		50	Gitaí et al., 2005
O. maracasense L.B.Sm.		150	Cotias-de-Oliveira et al., 2000
O. saxicola (Ule) L.B.Sm.		50	Ramiréz-Morillo and Brown, 2001;<br>Cotias-de-Oliveira et al., 2004
O. vagans M.B.Foster		50	Louzada et al., 2010
Portea K.Koch.			
P. grandiflora Philcox		50	Cotias-de-Oliveira et al., 2004
P. kermesina K.Koch		50	Weiss, 1965
P. petropolitana (Wawra) Mez		50	Gitaí et al., 2014

Pseudananas Hassl. ex Harms			
P. sagenarius (Arruda) Camargo [also published as *Pseudananas macrodontes* (E.Morren) Harms]		100<br>(98), 100	Lindschau, 1933<br>Lin et al., 1987
Quesnelia Gaudich.			
Q. arvensis (Vell.) Mez		50	Cotias-de-Oliveira et al., 2004; Gitaí et al., 2014
Q. edmundoi L.B.Smith var. *rubrobracteata* E.Pereira		50	Cotias-de-Oliveira et al., 2004
Q. liboniana (De Jonghe) Mez [also published as *Billbergia liboniana* De Jonghe, *Billbergia liboniana* D.Forst]	 <br>(54)	50	Sharma and Gosh, 1971; Brown et. al. 1997<br>Matsuura and Sutô, 1935
Sincoraea Ule			
Sincoraea albopicta (Philcox) Louzada & Wand. [published as *Orthophytum albopictum* Philcox]	 50	100	Cotias-de-Oliveira et al., 2004<br>Louzada et al., 2010
Wittmackia Mez			
W. lingulata (L.) Mez [published as *Aechmea lingulata* (L.) Baker var. *lingulata*]	≈25		Marchant, 1967
**PUYOIDEAE**			
**Taxon**	**n**	**2n**	**References**
Puya Molina			
P. alpestris (Poepp.) Gay		50	Marchant, 1967
P. chilensis Molina	(24)	 50	Tschischow, 1956<br>Marchant, 1967
P. raimondii Harms		50	Farvarger and Huynh, 1965

References

Bellintani, M.C., Assis, J.G.A., and Cotias-De-Oliveira, A.L.P. (2005). Chromosomal evolution of Bromeliaceae. Cytologia 70, 129-133. doi: 10.1508/cytologia.70.129

Brown, G.K., Varadarajan, G.S., and Gilmartin, A.J. (1984). Chromosome numbers reports LXXXV. Bromeliaceae. Taxon 33, 756-760.

Brown, G.K., Palací, C.A., and Luther, H. (1997). Chromosome numbers in Bromeliaceae. Selbyana 18, 85-88. doi: 10.2307/2444413

Ceita, G.O., Assis, J.G.A., Guedes, M.L.S., and Cotias-De-Oliveira, A.L.P. (2008). Cytogenetics of Brazilian species of Bromeliaceae. Bot. J. Linn. Soc. 158, 189-193. doi: 10.1111/j.1095-8339.2008.00776.x

Collins JL, Kerns KR. 1931. Genetic studies in pineapples. I. A preliminary report upon the chromosome number and meiosis in seven pineapples varieties. (*Ananas sativus* Lindl.) and in *Bromelia pinguin* L. J. Hered. 22, 139-142. doi: 10.1093/oxfordjournals.jhered.a103460

Cotias-de-Oliveira, A.L.P., Assis, J.G.A., Bellintani, M.C., Andrade, J.C., and Guedes, M.L.S. (2000). Chromosome numbers in Bromeliaceae. Genet. Mol. Biol. 23, 173-177. doi: 10.1590/S1415-47572000000100032

Cotias-de-Oliveira, A.L., Assis, J.G.A., Ceita, G.O., Palmeira, A.C.L., and Guedes, M.L.S. (2004). Chromosome numbers for Bromeliaceae species occurring in Brazil. *Cytologia* 69, 161-166. doi: 10.1508/cytologia.69.161

Éder-Silva, E., Pessoa-Felix, L., and Alcantara, B.R.L. 2007. Citogenética de algumas espécies frutíferas nativas do nordeste do Brasil. Rev. Bras. Frutic. 29, 110-114. Doi: 10.1590/S0100-29452007000100024

Favarger, C., and Huynh, K.L. (1965). „Bromeliaceae," in IOPB Chromosome Number Reports III, eds. Á. Löve, and O.T. Solbrig. Taxon 14, 52

Gitaí, J., Horres, R., and Benko-Iseppon, A.M. (2005). Chromosome features and evolution of Bromeliaceae. Plant Syst. Evol. 253, 65-80. doi: 10.1007/s00606-005-0306-8

Gitaí, J., Paule, J., Zizka, G., Schulte, K., and Benko-Iseppon, A.M.. (2014). Chromosome numbers and DNA content in Bromeliaceae: additional data and critical review. Bot. J. Lin. Soc. 176, 349-368. doi: 10.1111/boj.12211

Gouda, E.J., D. Butcher, C.S. Gouda. cont. updated. Encyclopaedia of Bromeliads, Version 4. Available online at: http://bromeliad.nl/encyclopedia

Lin, B.V., Ritschel, P.S., and Ferreira, F.R.. 1987. Número cromossômico de exemplares da família Bromeliaceae. Rev. Bras. Frutic. 9, 49-55.

Lindschau, M. (1933). Beiträge zur Zytologie der Bromeliaceae. Planta 3, 506-530. doi: 10.1007/BF01910644

Louzada, R.B., Palma-Silva, C., Corrêa, A.M., Kaltchuk-Silva, E., and Wanderley, M.G.L. (2010). Chromosome numbers of *Orthophytum* species (Bromeliaceae). Kew Bull. 65, 53-58. doi: 10.1007/s12225-010-9175-6

Marchant, C.J. (1967). Chromosome evolution in the Bromeliaceae. Kew Bull. 21, 161-168.

Matsuura, H., and Sutô, H. (1935). Contributions to the idiogram study in phanerogamous plants I. J. Fac. Sci., Hokkaido Univ. 5, 33-75.

Matuszak-Renger, S., Paule, J., Heller, S., Leme, E.M.C., Steinbeisser, G.M., Barfuss, M.H.J., et al. (2018). Phylogenetic relationships among *Ananas* and related taxa (Bromelioideae, Bromeliaceae) based on nuclear, plastid and AFLP data. Plant Syst. Evol. 304, 841-851. doi: 10.1007/s00606-018-1514-3

Palma-Silva, C. (2003). Análises citogenéticas em espécies de Vriesea e Aechmea (Bromeliaceae) nativas do Rio Grande do Sul. [Master thesis]. [Porto Alegre]: Universidade Federal do Rio Grande do Sul

Palma-Silva, C., Santos, D.G., Kaltchuk-Santos, E., and Bordanese-Zanettini, M.H. (2004). Chromosome numbers, meiotic behavior, and pollen viability of species of *Vriesea* and *Aechmea* genera (Bromeliaceae) native to Rio Grande do Sul, Brasil. Am. J. Bot. 91, 804-807. doi: 10.3732/ajb.91.6.804

Ramírez-Morillo, I.M., and Brown, G.K. (2001). The origin of the low chromosome number in Cryptanthus (Bromeliaceae). Syst. Bot. 26, 722-726. doi: 10.1043/0363-6445-26.4.722

Sharma, A.K., and Gosh, I. (1971). Cytotaxonomy of the family Bromeliaceae. Cytologia 36, 237-247. doi: 10.1508/cytologia.36.237

Taylor, W.R. (1925). Chromosome constrictions as distinguishing characteristics in plants. Am. J. Bot. 4, 238-244. doi: 10.1002/j.1537-2197.1925.tb05830.x

Tschischow, N.T. (1956). Número de cromosomas de algunas plantas chilenas. Bol. Soc. Biol. Concepc. 31, 145-147.

Weiss, H.E. (1965). Étude caryologique et cyto-taxonomique de quelques Broméliaceés. Mém. Mus. Natl. Hist. Nat. 1, 9-38.
