# Supplementary material for: Early Diverging and Core Bromelioideae (Bromeliaceae) Reveal Contrasting Patterns of Genome Size Evolution and Polyploidy
Source: Front Plant Sci. 2020 Sep 9;11:1295. doi: 10.3389/fpls.2020.01295 (PMC7509451; doi:10.3389/fpls.2020.01295)
Supplement: Supplementary file 7 [file Table_3.pdf]

Supplementary Table 3. Previously published genome size (2C-values), derived Cx values (based on the 2C threshold for diploids) and GC content estimates of the subfamily Bromelioideae and Puyoideae. Values marked with * were used for the analyses of this study. Nomenclature follows Gouda et al. (cont. updated) except the genus *Ananas* and *Pseudananas*.

**BROMELIOIDEAE**					
**Taxon**	**2C [pg]**	**SD**	**Derived Cx [pg]**	**GC content [%]**	**Reference**
Aechmea Ruiz & Pav.					
A. angustifolia Poepp. Endl.	0.69*	±0.005	0.345*		Müller et al. 2019
A. aquilega (Salisb.) Griseb	1.08*	±0.013	0.540*		Gitaí et al., 2014
A. bracteata (Sw.) Griseb.	0.81*	±0.005	0.405*		Müller et al. 2019
A. coelestis (K.Koch) E.Morren	0.96*	±0.004	0.480*		Müller et al. 2019
A. eurycorymbus Harms	2.19*	±0.013	0.5473*		Gitaí et al., 2014
A. fendleri André ex Mez	1.25*	±0.018	0.625*		Gitaí et al., 2014
A. filicaulis (Griseb.) Mez	1.67<br>1.77	±0.020<br>±0.002		44.00	Šmarda et al., 2014<br>Müller et al. 2019
A. fosteriana L.B.Sm.	0.93*	±0.001	0.465*		Müller et al. 2019
A. mexicana Baker	0.76	±0.009			Müller et al. 2019
A. nudicaulis (L.) Griseb. var. *cuspidata* Baker	0.78	±0.000		35.12	Favoreto et al., 2012
A. racinae L.B.Sm	0.88	±0.016			Müller et al. 2019
A. ramosa Mart. ex Schult.f. & Schult.f.	1.37*	±0.018	0.685*	38.63*	Favoreto et al., 2012
A. victoriana L.B.Sm.	0.92*	±0.002	0.460*		Müller et al. 2019
A. weilbachii Didr.	1.10*	±0.001	0.550*		Müller et al. 2019
Ananas Mill.					
A. bracteatus (Lindl.) Schult. & Schult.f.	0.92				Arumuganathan & Earle, 1991
A. comosus (L.) Merr.	1.09<br>0.93	 ±0.003			Arumuganathan & Earle, 1991<br>Müller et al. 2019
Billbergia Thunb**.**					
B. amoena (Lodd.) Lindl.	0.74	±0.002			Müller et al. 2019
B. brasiliensis L.B.Sm. [also published as *Billbergia velascana* Cárdenas]	0.91*<br>0.90	±0.005<br>±0.011	0.455*		Müller et al. 2019<br>Müller et al. 2019
B. euphemiae E.Morren	0.89*	±0.014	0.445*	37.42*	Favoreto et al., 2012
B. horrida Regel<br>*B. horrida* Regel var. *tigrina* hort. ex Baker	0.77*<br>0.85*	±0.005<br>±0.014	0.385*<br>0.425*	35.35	Favoreto et al., 2012<br>Gitaí et al., 2014
B. magnifica Mez	0.95*	±0.014	0.475*		Müller et al. 2019
B. nutans H.Wendl. ex Regel	0.7517	±0.0567			Ramírez-Morillo & Brown, 2001
B. pallidiflora Liebm.	1.03*	±0.009	0.515*		Gitaí et al., 2014
B. pyramidalis (Sims) Lindl.	0.90*	±0.003	0.450*		Müller et al. 2019
B. tweedieana Baker	0.95	±0.014		33.95	Favoreto et al., 2012
B. viridiflora H.Wendl.	0.66*	±0.002	0.330*		Müller et al. 2019
Bromelia L.					
B. antiacantha Bertol.	0.81*	±0.014	0.405*	38.43*	Favoreto et al., 2012
Canistropis Mez					
C. billbergioides (Schult.f.) Leme [also published as *Nidularium billbergioides* (Schult. & Schult.f.) L.B.Sm.]	1.00<br>1.02	±0.005		37.36	Favoreto et al., 2012<br>Müller et al. 2019
Cryptanthus Otto & A.Dietr.					
C. acaulis (Lindl.) Beer	1.38*	±0.0283			Ramírez-Morillo & Brown, 2001

	1.38				Cruz et al., 2020
C. alagoanus Leme & J.A.Siqueira	0.78				Cruz et al., 2020
C. arelii H.Luther	0.98				Cruz et al., 2020
C. bahianus L.B.Sm.	0.75	±0.0683			Ramírez-Morillo & Brown, 2001
C. beuckeri E.Morren	1.458*<br>1.46	±0.0179			Ramírez-Morillo & Brown, 2001<br>Cruz et al., 2020
C. bromelioides Otto & A.Dietr.	1.52				Cruz et al., 2020
C. burle-marxii Leme	1.32				Cruz et al., 2020
C. capitellatus Leme & L.Kollmann	1.36				Cruz et al., 2020
C. colnagoi Rauh & Leme	1.34				Cruz et al., 2020
C. coriaceus Leme	0.84				Cruz et al., 2020
C. correia-araujoi Leme	1.66				Cruz et al., 2020
C. delicatus Leme	1.46				Cruz et al., 2020
C. dianae Leme	1.36				Cruz et al., 2020
C. giganteus Leme & A.P.Fontana	1.38				Cruz et al., 2020
C. lutherianus I.Ramirez	0.90				Cruz et al., 2020
C. lyman-smithii Leme	1.38				Cruz et al., 2020
C. marginatus L.B.Sm.	1.26*<br>1.40	±0.005			Gitaí et al., 2014<br>Cruz et al., 2020
C. maritimus L.B.Sm.	1.48				Cruz et al., 2020
C. pickelii L.B.Sm.	1.28				Cruz et al., 2020
C. praetextus E.Morren ex Baker	1.35*	±0.014			Gitaí et al., 2014
C. reisii Leme	0.88				Cruz et al., 2020
C. sinuosus L.B.Sm.	1.04				Cruz et al., 2020
C. teretifolius Leme	1.40				Cruz et al., 2020
C. ubairensis I.Ramirez	1.00				Cruz et al., 2020
C. venecianus Leme & L.Kollmann	1.32				Cruz et al., 2020
C. viridipetalus Leme	1.34				Cruz et al., 2020
C. zonatus (Vis.) Beer	1.04				Cruz et al., 2020
C. sp.	1.33*	±0.015		38.78*	Favoreto et al., 2012
Edmundoa Leme					
E. lindenii (Regel) Leme	1.32*	±0.010	0.662*		Gitaí et al., 2014
Fascicularia Mez					
F. bicolor (Ruiz & Pav.) Mez ssp. *bicolor*<br>*F. bicolor* (Ruiz & Pav.) Mez ssp. *canaliculata* E.C.Nelson & Zizka	1.05*<br>1.12*	±0.010<br>±0.008	0.527*<br>0.560*		Gitaí et al., 2014<br>Gitaí et al., 2014
Forzzaea Leme, S. Heller & Zizka					
F. leopolodo-horstii Leme, S.Heller & Zizka	1.14				Cruz et al., 2020
Greigia Regel					
G. sphacelata (Ruiz & Pav.) Regel	1.56*	±0.004	0.779*		Gitaí et al., 2014
G. sp.	1.35*	±0.019	0.664*		Gitaí et al., 2014
Hohenbergia Schult. & Schult.f.					
H. aechmeoides Leme [published as *Hohenberiga aechmeoides* Leme]	1.48				Gonçalves-Oliveira et al., 2020
H. arcuata Leme & M.Machado	1.42				Gonçalves-Oliveira et al., 2020
H. augusta (Vell.) E.Morren [published as *Hohenbergia augusta* Mez]	1.23<br>1.24				Gonçalves-Oliveira et al., 2020
H. barbarespina Leme & Fraga	1.37				Gonçalves-Oliveira et al., 2020
H. belemii L.B.Sm & Read	1.32				Gonçalves-Oliveira et al., 2020
H. blanchetii (Baker) Mez [published as *Hohenbergia blanchetti* É.Mooren]	1.40				Gonçalves-Oliveira et al., 2020
H. burle-marxii Leme & W.Till	1.39				Gonçalves-Oliveira et al., 2020
H. capitata Schult. & Schult.f. [published as *Hohenbergia capitata* Schult.f.]	1.50				Gonçalves-Oliveira et al., 2020

H. castellanosii L.B.Sm. & Read	1.19<br>1.23<br>1.24				Gonçalves-Oliveira et al., 2020
H. catingae Ule	1.29<br>1.41<br>1.42<br>1.48				Gonçalves-Oliveira et al., 2020
H. edmundoi L.B.Sm & Read	1.36				Gonçalves-Oliveira et al., 2020
H. flava Leme & C.C.Paula	1.39				Gonçalves-Oliveira et al., 2020
H. horrida Harms	1.17				Gonçalves-Oliveira et al., 2020
H. igatuensis Leme	1.41				Gonçalves-Oliveira et al., 2020
H. lanata E.Pereira & Moutinho	1.46				Gonçalves-Oliveira et al., 2020
H. lativaginata J.R.Maciel & Louzada	1.22				Gonçalves-Oliveira et al., 2020
H. lemei H.Luther & K.Norton	1.32				Gonçalves-Oliveira et al., 2020
H. leopoldo-horstii E.Gross, Rauh & Leme	1.333				Gonçalves-Oliveira et al., 2020
H. littoralis L.B.Sm.	0.78				Gonçalves-Oliveira et al., 2020
H. loredanoana Leme & L.Kollmann	1.30				Gonçalves-Oliveira et al., 2020
H. magnispina Leme	1.27				Gonçalves-Oliveira et al., 2020
H. minor L.B.Sm.	1.28				Gonçalves-Oliveira et al., 2020
H. pennae E.Pereira	1.39				Gonçalves-Oliveira et al., 2020
H. ramageana Mez	1.42				Gonçalves-Oliveira et al., 2020
H. reconcavensis Leme & Fraga	1.35				Gonçalves-Oliveira et al., 2020
H. ridleyi (Baker) Mez	1.34				Gonçalves-Oliveira et al., 2020
H. rosea L.B.Sm & Read	1.33				Gonçalves-Oliveira et al., 2020
H. stellata Schult. & Schult.f.	1.33<br>1.34<br>1.36				Gonçalves-Oliveira et al., 2020
H. utriculosa Ule	1.52				Gonçalves-Oliveira et al., 2020
H. vestita L.B.Sm.	1.17				Gonçalves-Oliveira et al., 2020
Hoplocryptanthus (Mez) Leme, S. Heller & Zizka					
H. caracensis Leme, S.Heller & Zizka	0.78				Cruz et al., 2020
H. ferrarius Leme, S.Heller & Zizka	0.82				Cruz et al., 2020
H. glaziovii Leme, S.Heller & Zizka	0.80				Cruz et al., 2020
H. regius Leme, S.Heller & Zizka	0.78				Cruz et al., 2020
H. schwackeanus (Mez) Leme, S. Heller & Zizka [published as *Cryptanthus schwackeanus* Mez]	0.71*	±0.0141			Ramírez-Morillo & Brown, 2001
H. schwackeanus (Mez) Leme, S.Heller & Zizka	0.82				Cruz et al., 2020
H. tiradentesensis Leme, S.Heller & Zizka	0.80				Cruz et al., 2020
Neoregelia L.B.Sm.					
N. carolinae (Beer) L.B.Sm	0.96*	±0.028	0.480*		Müller et al. 2019
N. aff. *simulans* L.B.Sm.	0.98*	±0.004	0.490*	37.24*	Favoreto et al., 2012
N. zonata L.B.Sm.	0.93*	±0.001	0.465*		Müller et al. 2019
***Nidularium* Lem.**					
N. camposportoi (L.B.Sm.) Wand. & B.A.Moreira [published as *Nidularium campos-portoi* (L.B.Sm.) Leme]	1.03*	±0.003	0.515*		Müller et al. 2019
N. innocentii Lem.	1.12*	±0.005	0.560*		Müller et al. 2019
Ochagavia Phil.					
O. elegans Phil.	1.12*	±0.015	0.557*		Gitaí et al., 2014
Orthophytum Beer					
O. foliosum L.B.Sm.	1.88*		0.313*		Müller et al. 2019
O. saxicola (Ule) L.B.Sm.	0.64*	±0.0141	0.320*		Ramírez-Morillo & Brown, 2001

Portea K.Koch.					
P. petropolitana (Wawra) Mez	1.31*	±0.011	0.655*		Gitaí et al., 2014
Pseudananas Hassl. ex Harms					
P. sagenarius (Arruda) Camargo [published as *Ananas macrodontes* E.Morren]	1.00	±0.021		39.24	Favoreto et al., 2012
Quesnelia Gaudich.					
Q. arvensis (Vell.) Mez	0.88	±0.001			Gitaí et al., 2014
Rokautskyia Leme, S. Heller & Zizka					
R. latifolia Leme, S.Heller & Zizka	1.10				Cruz et al., 2020
R. microglazioui Leme, S.Heller & Zizka	0.94				Cruz et al., 2020
R. odoratissima Leme, S.Heller & Zizka	1.10				Cruz et al., 2020
R. pseudoglasioui Leme, S.Heller & Zizka	1.00				Cruz et al., 2020
R. pseudoscaposa Leme, S.Heller & Zizka	1.04				Cruz et al., 2020
R. sanctaluciae Leme, S.Heller & Zizka	1.00				Cruz et al., 2020
R. scaposa Leme, S.Heller & Zizka	1.04				Cruz et al., 2020
R. whitmanii Leme, S.Heller & Zizka	1.06				Cruz et al., 2020
Ronnbergia					
R. deleonii L.B.Sm.	1.38*	±0.024			Müller et al. 2019
R. veitchii (Baker) Aguirre-Santoro [published as *Aechmea veitchii* Baker]	0.77*	±0.008	0.385*		Müller et al. 2019
R. weberbaueri (Harms) Aguirre-Santoro [published as *Aechmea weberbaueri* Harms]	1.32	±0.008			Müller et al. 2019
R. wuelfinghoffii (E.Gross.) Aguirre-Santoro [published as *Aechmea wuelfinghoffii* E.Gross]	1.03*	±0.002	0.515*		Müller et al. 2019
Sincoraea Ule					
S. amoena Leme, S.Heller & Zizka	1.04				Cruz et al., 2020
S. heleniceae Leme, S.Heller & Zizka	1.08				Cruz et al., 2020
S. ophiuroides Leme, S.Heller & Zizka	1.02				Cruz et al., 2020
S. ulei Leme, S.Heller & Zizka	1.50				Cruz et al., 2020
xHohenmea B.R.Silva & L.F.Sousa					
xH. itaipuana B.R.Silva & L.F.Sousa	1.63				Gonçalves-Oliveira et al., 2020
**PUYOIDEAE**					
Puya Molina					
P. coerulea Miers	1.02	±0.013			Gitaí et al., 2014
P. mirabilis (Mez) L.B.Sm.	0.88<br>0.99	 ±0.010			Ebert & Till, 1997<br>Gitaí et al., 2014
P. raimondii Harms	1.13*				Sgorbati et al., 2007
P. stenothyrsa (Baker) Mez	0.94				Ebert & Till, 1997

References

Arumuganathan, K., and Earle, E.D. (1991). Nuclear DNA content of some important plant species. Plant Mol. Biol. Rep. 9: 208-218. doi: 10.1007/BF02672069

Cruz, G.A.S, Mendonça Filho, J.R., Vasconcelos, S., Gitaí, J., Salabert De Campos, J.M., Facio Viccini, L., et al. (2020). Genome size evolution and chromosome numbers of species of the

cryptanthoid complex (Bromelioideae, Bromeliaceae) in a phylogenetic framework. Bot. J. Linn. Soc. 192, 887-899. doi: 10.1093/botlinnean/boz103

Ebert, I., and Till, W. (1997). “Nuclear genome size in Pitcairnioideae (Bromeliaceae) with emphasis on the genus *Pitcairnia,”* in Abstracts, Angiosperm genome size discussion meeting, ed. Anonymous (Kew: Royal Botanical Gardens).

Favoreto, F.C., Carvalho, C.R., Lima, A.B.P., Ferreira, A., and Clarindo, W.R. (2012). Genome size and base composition of Bromeliaceae species assessed by flow cytometry. Plant Syst. Evol. 298, 1185-1193. doi: 10.1007/s00606-012-0620-x

Gitaí, J., Paule, J., Zizka, G., Schulte, K., and Benko-Iseppon, A.M.. (2014). Chromosome numbers and DNA content in Bromeliaceae: additional data and critical review. Bot. J. Lin. Soc. 176, 349-368. doi: 10.1111/boj.12211

Gonçalves-Oliveira, R.C., Ximenes, A.F., Oliveira, A.R., Vasconcelos, S., Carvalho-Filho, N., Oliveira, G., et al. (2020). Genome size and chromosome number conservation contrasting with karyotype diversity in *Hohenbergia* (Bromelioideae, Bromeliaceae). Bot. J. Linn. Soc. 192, 900-909. doi: 10.1093/botlinnean/boz094

Gouda, E.J., D. Butcher, C.S. Gouda. cont. updated. Encyclopaedia of Bromeliads, Version 4. Available online at: http://bromeliad.nl/encyclopedia

Müller, L.L.B., Zotz, G., and Albach, D.C. (2019). Bromeliaceae subfamilies show divergent trends of genome size evolution. Sci. Rep. 9, 5136. doi: 10.1038/s41598-019-41474-w

Ramírez-Morillo, I.M., and Brown, G.K. (2001). The origin of the low chromosome number in *Cryptanthus* (Bromeliaceae). Syst. Bot. 26, 722-726. doi: 10.1043/0363-6445-26.4.722

Šmarda, P., Bureš, P., Horová, L., Leitch, I.J., Mucina, L., Pacini, E., et al. (2014). Ecological and evolutionary significance of genomic GC content diversity in monocots. Proc. Natl. Acad. Sci. U.S.A. 111, E4096-E4102. doi: 10.1073/pnas.1321152111
