# Supplementary material for: Early Diverging and Core Bromelioideae (Bromeliaceae) Reveal Contrasting Patterns of Genome Size Evolution and Polyploidy
Source: Front Plant Sci. 2020 Sep 9;11:1295. doi: 10.3389/fpls.2020.01295 (PMC7509451; doi:10.3389/fpls.2020.01295)
Supplement: Supplementary file 8 [file Table_4.pdf]

SupplementaryTable 4. Correlation coefficients between 19 climatic variables extracted for the distribution ranges of studied Bromelioideae species. Bold font highlights the absolute values greater than 0.75. Variables in bold were omitted in the PCA as well as regression analyses.

	**Bio1**	Bio2	**Bio3**	Bio4	Bio5	Bio6	**Bio7**	**Bio8**	**Bio9**	**Bio10**	**Bio11**	**Bio12**	Bio13	Bio14	**Bio15**	**Bio16**	**Bio17**	Bio18	**Bio19**
Bio1	X																		
Bio2	-0.412	X																	
Bio3	-0.412	-0.024	X																
Bio4	0.372	0.276	**-0.882**	X															
Bio5	-0.548	0.227	-0.194	-0.061	X														
Bio6	0.184	-0.576	0.447	-0.705	0.277	X													
Bio7	**0.928**	**0.761**	-0.556	**0.805**	-0.044	**-0.825**	X												
Bio8	-0.593	-0.279	0.143	-0.268	0.180	**0.760**	-0.335	X											
Bio9	**0.899**	-0.486	0.495	-0.693	0.176	**0.937**	-0.733	0.689	X										
Bio10	**0.929**	-0.397	0.099	-0.242	0.130	**0.795**	-0.378	**0.934**	**0.804**	X									
Bio11	**0.941**	-0.414	0.540	-0.727	0.207	**0.965**	-0.716	**0.809**	**0.958**	**0.837**	X								
Bio12	**0.971**	-0.282	0.436	-0.429	-0.056	0.388	-0.412	0.212	0.381	0.220	0.382	X							
Bio13	0.308	0.097	0.233	-0.297	0.421	0.251	-0.151	0.108	0.245	0.081	0.251	**0.751**	X						
Bio14	0.174	-0.452	0.269	-0.367	0.095	0.432	-0.537	0.166	0.345	0.182	0.337	**0.779**	0.411	X					
Bio15	0.268	0.617	-0.089	0.230	-0.011	-0.401	0.566	-0.161	-0.256	-0.179	-0.255	-0.414	0.036	**-0.795**	X				
Bio16	-0.219	-0.002	0.352	-0.289	-0.047	0.192	-0.127	0.143	0.242	0.139	0.247	**0.876**	**0.871**	0.422	0.035	X			
Bio17	0.194	-0.469	0.295	-0.384	0.071	0.447	-0.556	0.175	0.364	0.193	0.353	**0.803**	0.425	**0.996**	**-0.797**	0.447	X		
Bio18	0.282	0.054	-0.001	0.100	-0.078	-0.130	0.122	0.048	-0.229	-0.097	-0.132	0.635	0.523	0.443	-0.139	0.640	0.443	X	
Bio19	-0.132	-0.496	0.427	-0.525	0.025	0.525	-0.631	0.128	0.555	0.245	0.463	**0.815**	0.553	**0.774**	-0.543	0.595	**0.801**	0.184	X

Bio1 Annual mean temperature
Bio2 Mean diurnal range
Bio3 Isothermality
Bio4 Temperature seasonality
Bio5 Max temperature of the warmest month
Bio6 Min temperature of the coldest month
Bio7 Temperature annual range
Bio8 Mean temperature of the wettest quarter
Bio9 Mean temperature of the driest quarter
Bio10 Mean temperature of the warmest quarter
Bio11 Mean temperature of the coldest quarter
Bio12 Annual precipitation
Bio13 Precipitation of the wettest month
Bio14 Precipitation of the driest month
Bio15 Precipitation seasonality
Bio16 Precipitation of the wettest quarter
Bio17 Precipitation of the driest quarter
Bio18 Precipitation of the warmest quarter
Bio19 Precipitation of the coldest quarter
