# Supplementary material for: Early Diverging and Core Bromelioideae (Bromeliaceae) Reveal Contrasting Patterns of Genome Size Evolution and Polyploidy
Source: Front Plant Sci. 2020 Sep 9;11:1295. doi: 10.3389/fpls.2020.01295 (PMC7509451; doi:10.3389/fpls.2020.01295)
Supplement: Supplementary file 9 [file Table_5.pdf]

Supplementary Table 5. Performance of Ornstein–Uhlenbeck (OU) and Brownian motion (BM) models of genomic character evolution (2C, Cx, GC content) among early diverging and core Bromelioideae. Bold font indicates the best fitting model. Models with no values ("-") failed to pass the model performance criteria.

Model	lnL	AIC	AICc	AICc weight
**2C**				
BM1	-62.5161	129.0322	129.1547	0.0000
**BMS**	**-23.1937**	**52.3874**	**52.6348**	**0.8901**
OU1	-50.2777	106.5554	106.8028	0.0000
OUM	-49.8493	107.6985	108.1152	0.0000
OUMA	-	-	-	-
OUMV	-23.0935	56.1871	56.8187	0.1099
OUMVA	-30.9180	73.8361	74.7297	0.0000
**Cx**				
BM1	18.26014	-32.52028	-32.38392	0.0000
BMS	30.47079	-54.94158	-54.66572	0.0000
OU1	60.96096	-115.9219	-115.6461	0.0062
OUM	61.24457	-114.4891	-114.024	0.0028
OUMA	-	-	-	-
**OUMV**	**68.2466**	**-126.4932**	**-125.7873**	**0.9910**
OUMVA	-	-	-	-
**GC**				
BM1	-146.1360	296.2720	296.3970	0.0000
BMS	-132.1206	270.2412	270.4938	0.0000
**OU1**	**-117.6816**	**241.3632**	**241.6158**	**0.6052**
OUM	-117.3284	242.6569	243.0824	0.2907
OUMA	-	-	-	-
OUMV	-117.2455	244.4909	245.1361	0.1041
OUMVA	-	-	-	-
