# Supplementary material for: Early Diverging and Core Bromelioideae (Bromeliaceae) Reveal Contrasting Patterns of Genome Size Evolution and Polyploidy
Source: Front Plant Sci. 2020 Sep 9;11:1295. doi: 10.3389/fpls.2020.01295 (PMC7509451; doi:10.3389/fpls.2020.01295)
Supplement: Supplementary file 10 [file Table_6.pdf]

Supplementary Table 6. Overview of multiple regression and multiple phylogenetic generalized least squares (PGLS) analyses of genomic characters and bioclimatic variables in studied Bromelioideae. Bold font indicates significant associations after Bonferroni correction (p_i^*-value).

**multiple regression**	**Regression Slope Estimate**	**Standard error**	**t-test statistic**	**p-value**	**p_i^*-value**
2C ~ bio02	-0.0938542	0.0885182	-1.060	0.2906	2.0342
2C ~ bio04	-0.0013134	0.0022477	-0.584	0.5598	3.9186
2C ~ bio05	0.1062077	0.0837800	1.268	0.2067	1.4469
2C ~ bio06	-0.0962120	0.0810294	-1.187	0.2368	1.6576
2C ~ bio13	-0.0006685	0.0005510	-1.213	0.2267	1.5869
2C ~ bio14	0.0008622	0.0013000	0.663	0.5081	3.5567
2C ~ bio18	-0.0005195	0.0002698	-1.925	0.0559	0.3913
Multiple R^2: 0.1165 p-value: 0.004					
**multiple PGLS**	**Regression Slope Estimate**	**Standard error**	**t-test statistic**	**p-value**	**p_i^*-value**
2C ~ bio02	0.0789	0.1089	0.7244	0.4709	3.2963
2C ~ bio04	0.0027	0.0027	0.9995	0.3205	2.2438
2C ~ bio05	-0.0587	0.1023	-0.5741	0.5675	3.9725
2C ~ bio06	0.0773	0.0982	0.7865	0.4338	3.0369
2C ~ bio13	-0.0006	0.0007	-0.8020	0.4249	2.9741
2C ~ bio14	-0.0013	0.0015	-0.8659	0.3891	2.7238
2C ~ bio18	-0.0001	0.0004	-0.3551	0.7234	5.0641
λ (ML) : 0.931 95.0% CI : (0.831, 0.980)<br>Multiple R^2: 0.0819 p-value: 0.4156					
**Cx ~ bio02**	**7.36E-02**	**1.89E-02**	**3.9029**	**0.0002**	**0.0015**
**Cx ~ bio04**	**1.43E-03**	**4.65E-04**	**3.0775**	**0.0030**	**0.0207**
**Cx ~ bio05**	**-6.68E-02**	**1.71E-02**	**-3.9150**	**0.0002**	**0.0014**
**Cx ~ bio06**	**5.76E-02**	**1.61E-02**	**3.5812**	**0.0006**	**0.0043**
Cx ~ bio13	9.82E-05	1.41E-04	0.6988	0.4869	3.4085
Cx ~ bio14	-1.10E-04	2.85E-04	-0.3864	0.7003	4.9023
Cx ~ bio18	-3.48E-05	6.69E-05	-0.5199	0.6047	4.2332
λ (ML) : 0.998 95.0% CI : (0.966, NA)<br>Multiple R^2: 0.2026 p-value: 0.0184					
**GC ~ bio02**	**0.7703**	**0.2221**	**3.4684**	**0.0008**	**0.0059**
GC ~ bio04	0.0123	0.0057	2.1693	0.0330	0.2311
**GC ~ bio05**	**-0.7687**	**0.2102**	**-3.6573**	**0.0004**	**0.0032**
**GC ~ bio06**	**0.5837**	**0.2034**	**2.8698**	**0.0052**	**0.0368**
GC ~ bio13	-0.0002	0.0014	-0.1532	0.8786	6.1506
GC ~ bio14	-0.0004	0.0029	-0.1671	0.8677	6.0741
GC ~ bio18	0.0007	0.0007	1.0560	0.2941	2.0589
λ (ML) : 0.786 95.0% CI : (NA, 0.952)<br>Multiple R^2: 0.2737 p-value: 0.0004					
